# Bioaccessibility and Antioxidant Activity of Faba Bean Peptides in Comparison to those of Pea and Soy after In Vitro Gastrointestinal Digestion and Transepithelial Transport across Caco-2 and HT29-MTX-E12 Cells

**DOI:** 10.1021/acs.jafc.4c02948

**Published:** 2024-08-01

**Authors:** Delphine Martineau-Côté, Allaoua Achouri, Mélanie Pitre, Salwa Karboune, Lamia L’Hocine

**Affiliations:** † Agriculture and Agri-Food Canada, Saint-Hyacinthe Research and Development Centre, Saint-Hyacinthe, Quebec J2S 8E3, Canada; ‡ Department of Food Science and Agricultural Chemistry, Macdonald Campus, McGill University, Sainte-Anne-de-Bellevue, Quebec H9X 3 V9, Canada

**Keywords:** Vicia faba L., in vitro gastrointestinal digestion, bioactive peptides, antioxidant, in vitro transepithelial transport, Caco-2, HT29-MTX, peptidomics

## Abstract

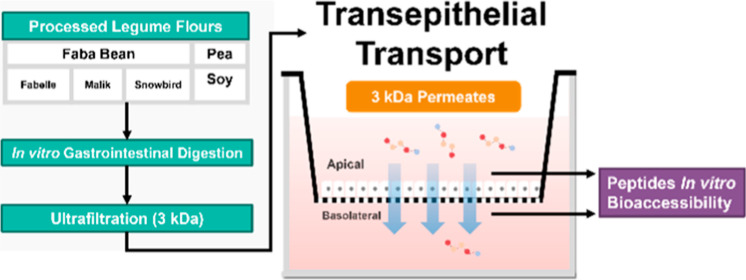

In this study, the transepithelial transport of bioactive peptides derived from faba bean flour gastrointestinal digestates was investigated, in vitro, using a Caco-2 and HT29-MTX-E12 coculture monolayer, in comparison to those of pea and soy. The profile of transported peptides was determined by mass spectrometry, and the residual antioxidant activity was assessed. The ORAC value significantly (*p* < 0.05) decreased after transepithelial transport (24–36% reduction) for all legumes, while the antioxidant activity in ABTS assay significantly (*p* < 0.05) increased, as shown by the EC_50_ decrease of 26–44%. Five of the nine faba bean peptides that crossed the intestinal cell monolayer exhibited antioxidant activity. Two of these peptides, TETWNPNHPEL and TETWNPNHPE, were further hydrolyzed by the cells’ brush border peptidases to smaller fragments TETWNPNHP and TWNPNHPE. These metabolized peptides were synthesized, and both maintained high antioxidant activity in both ABTS (EC_50_ of 1.2 ± 0.2 and 0.4 ± 0.1 mM, respectively) and ORAC (2.5 ± 0.1 and 3.4 ± 0.2 mM of Trolox equivalent/mM, respectively) assays. These results demonstrated for the first time the bioaccessibility of faba bean peptides produced after in vitro gastrointestinal digestion and how their bioactive properties can be modulated during transepithelial transport.

## 1 Introduction

Faba bean (*Vicia faba* L.) is a promising, high-quality, and sustainable protein source. Its high protein content (30%) and well-balanced amino acid composition make it a suitable protein source to be included in various food products.^1^ Besides its high nutritional value, faba bean has a high bioactive potential, being a rich source of bioactive compounds, including polyphenols, l-DOPA, γ-aminobutyric acid, resistant starch, fibers, and bioactive peptides.^1^ Food-derived bioactive peptides are promising health-promoting agents in the management of noncommunicable diseases.^2^ Faba bean protein hydrolysates with antioxidant, antidiabetic antihypertensive, cholesterol-lowering, anticancer, anti-inflammatory, immune-modulating, and food intake regulation properties have been identified to date.^1,3^

In a previous study,^4^ we have demonstrated that faba bean peptides generated after in vitro gastrointestinal digestion have a high bioactive potential, particularly in terms of antihypertensive and antioxidant activity. These bioactive properties were revealed to be associated with the release of bioactive peptides after in vitro gastrointestinal digestion.^3^ Among these, 7 novel faba bean-derived peptides (NYDEGSEPR, TETWNPNHPEL, TETWNPNHPE, VIPTEPPH, VIPTEPPHA, VVIPTEPPHA, and VVIPTEPPH) with antioxidant activity were identified. However, for these peptides to be active in vivo and exert their health beneficial effect, they need to be transported across the intestinal barrier and reach the bloodstream. It is now accepted that food-derived di- and tripeptides can be absorbed intact in the small intestine,^5^ and some of them were shown to exert health benefits in humans. Based on that, it is necessary to investigate the permeability potential of these faba bean-derived bioactive oligopeptides to anticipate their bioactive potential in vivo.

While human and animal models are the most significant way to assess bioactive peptide absorption and bioavailability, they pose ethical and economical limitations. Cellular models present a viable alternative, as they mimic the intestinal barrier without these drawbacks. To achieve this, intestinal cells are cultivated on a permeable support to mimic the intestinal barrier. The peptides of interest are added to the apical (AP) side, representing the intestinal lumen, and the in vitro bioaccessibility peptides are recovered in the basolateral (BL) side, representing the intestinal circulation. Caco-2 cells are preferred and most widely used to model the human intestinal barrier. Although there are still ongoing efforts to standardize and harmonize the cell-culture conditions,^6^ this model has proven its utility over the years as a first screening tool for permeability assessment. These cells differentiate spontaneously into enterocyte-like cells when they reach confluence.^7^ The cells form a polarized intestinal monolayer, which expresses tight junctions, microvilli, brush border digestive enzymes,^7^ and peptide transporters such as peptide transporter 1 (PepT1).^8^ The Caco-2 model can be further improved by the cocultivation of HT29-MTX-E12 cells to mimic the goblet-cell, the second most abundant cell type in the human small intestine.^9^ These cells produce mucins, which are highly glycosylated proteins that form a gel in the presence of water and act as a physical barrier in the intestine.^10^ This mucus layer limits particle diffusion and is therefore an essential element to consider when studying peptide transport.^11^

Data on the bioavailability of faba bean peptides after gastrointestinal digestion are not available. In an effort to help closing this research gap, we investigated the transepithelial transport of faba bean peptides after in vitro gastrointestinal digestion across a Caco-2 and HT29-MTX-E12 coculture monolayer and its impact of the modulation of their bioactive properties. The in vitro bioaccessibility of peptides from three faba bean cultivars (Fabelle, Malik, and Snowbird) was compared to that of two conventional legumes pea (Amarillo) and soy (AAC-26-15). We hypothesized that faba bean, pea, and soy peptides released by in vitro gastrointestinal digestion can be transported across intestinal cell monolayers while maintaining their antioxidant activities. We also expected differences among the different faba bean cultivars, pea, and soy because of their different protein composition and quality traits.

## 2 Materials and Methods

### 2.1 Chemicals

Three dehulled faba bean (FB) cultivars (FB-Fabelle, FB-Malik, and FB-Snowbird), one dehulled pea cultivar (Pea-Amarillo), and one dehulled soy cultivar (Soy-AAC-26-15) were used in this study. FB-Fabelle and FB-Malik cultivars were provided by AGT Foods and Ingredients (Saskatoon, SK, CA) and FB-Snowbird by W.A. Grain & Pulse Solutions (Innisfail, AB, CA). Certified yellow pea (CDC Amarillo) and soybean (Cdn no. 1, Variety AAC 26-25, Non-GMO & IP, Lot 261510504AT) were provided by Greenleaf Seeds (Tisdale, SK, CA) and Huron seeds (Clinton, ON, CA), respectively. Faba bean and pea samples were supplied as milled flours and soybean as whole seeds. Soy flour was prepared as previously described.^4^ For cell culture, Dulbecco’s modification of Eagle’s medium (DMEM) containing 4.5 g/L glucose, with phenol-red and without sodium pyruvate, 200 mM l-glutamine, Dulbecco’s phosphate-buffered-saline (D-PBS) without Ca^2+^ and Mg^2+^, D-PBS with Ca^2+^ and Mg^2+^, nonessential amino acid solution 100×, heat-inactivated fetal bovine serum (FBS), 5000 IU penicillin, 5000 μg/mL streptomycin solution, and trypsin solution (0.05%) containing 0.53 mM EDTA in HBSS were purchased from Wisent Bioproducts (Saint-Jean-Baptiste, QC, Canada). Hank’s Balanced Salt Solution (HBSS) was purchased from Gibco (product no. 14025092, Thermo Fisher Scientific, San Jose, CA, USA). Caco-2 cells (ATCC HTB-37, passage 18 when purchased) were procured from ATCC (Manassas, Virginia, USA). HT29-MTX-E12 cells (passage 50 when purchased) were obtained from the European Collection of Authenticated Cell Cultures (ECACC). Twelve mm Transwell with a 0.4 μm pore polyester membrane insert (Corning 3460), Alcian Blue 8GX, 4-nitrophenol solution 10 mM, *p*-nitrophenyl phosphate tablets, and Lucifer yellow CH dipotassium salt were purchased from Sigma-Aldrich (St. Louis, MO, USA).

The peptides derived from faba bean flour gastrointestinal digestate, TETWNPNHPEL and TETWNPNHPE, and their metabolites, TETWNPNHP and TWNPNHPE, were synthesized by Biomatik (Kitchener, Ontario, Canada). Their purity (>98%) and quality were checked by reverse-phase HPLC and mass spectrometry analysis.

The Pierce BCA Assay kit was purchased from Thermo Fisher Scientific (San Jose, CA, USA), and the Cell Titer-Glo 2.0 kit was purchased from Promega (Fitchburg, Wi, USA). All chemicals and reagents used were of analytical grade. Deionized water was used in all of the experiments.

### 2.2 Processing and In Vitro Gastrointestinal Digestion of Faba Bean, Pea, and Soy Flours

Before in vitro gastrointestinal digestion, the legume flours were thermally treated through boiling as previously described.^4^ Legume flours were then digested in vitro using the standardized INFOGEST protocol^12^ with modifications to include a jejunal-ileal digestion phase^13^ and ensure biocompatibility with cell culture.^4,13^ 1.2 g portion of legume flour was mixed with 1.8 g of water, and the suspension was mixed in a ratio 1:1 with digestive fluid to perform the digestion. Oral, gastric, duodenal, and jejunal-ileal digestion phases were performed as previously described.^4,13^ A digestion blank was omitted in this study because of the increased enzyme autolysis occurring in the absence of a protein substrate,^14^ leading to a peptide pool that is not representative of the digestive enzyme contribution in the presence of a protein substrate. It was hypothesized that the enzyme contribution was similar for all investigated legumes.

**Figure 1:**
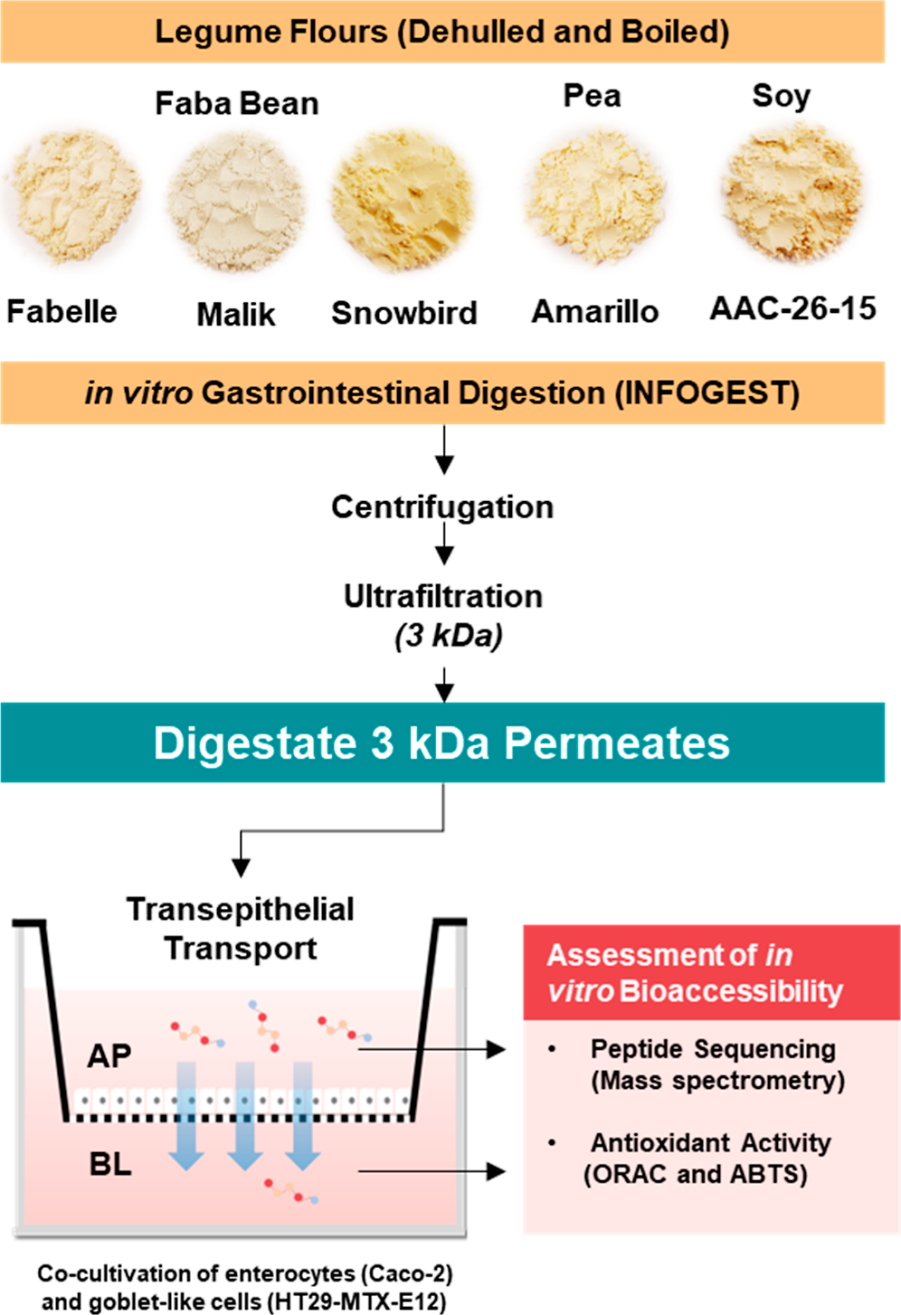
Experimental work flowchart.

At the end of the digestion, samples were cooled on ice, and a protease inhibitor (1 mM AEBSF) was added to inhibit trypsin and chymotrypsin activity. The digestates were centrifuged (15,000*g* for 30 min at 4 °C) to recover the soluble fraction. Potentially bioaccessible peptides were recovered through ultrafiltration on a 3 kilo Dalton (kDa) molecular weight cutoff membrane to remove residual active proteases and peptidases.^4^ The addition of AEBSF as a protease inhibitor and the ultrafiltration step, although potentially affecting the physiological correspondence of the model, were selected as a relevant strategy to reduce residual active proteases and peptidases, without modifying the structure of the small bioactive molecules of interest, as heat deactivation would do. The permeates of three independent digestions of legume flours were pooled together for the cell transport study. Pooled permeates’ osmolality was measured using a Micro-Osmometer (model 3320, Advanced Instruments INC., Norwood, Massachusetts), and permeates were diluted in water to reach a physiological osmolality of 285–300 mOsm/kg, and their pH was adjusted to a physiological value of 7.3. Protein content in the diluted permeates was determined with the Pierce BCA protein assay kit using bovine serum albumin as a standard. The 3 kDa permeates were frozen at −80 °C until the transport experiment. A flowchart representing the experimental protocol is presented in Figure 1.

### 2.3 Cell Culture

Caco-2 and HT29-MTX-E12 cells were cultivated separately in a growth medium, which was composed of DMEM supplemented with 10% FBS, 100 U/mL penicillin and 100 μg/mL streptomycin, 1% nonessential amino acids, and 2 mM l-glutamine and incubated at 37 °C in an atmosphere containing 5% CO_2_. Cells were subcultivated once a week at 80–90% confluence using a trypsin–EDTA solution, and the culture medium was changed every 2–3 days. Three passages were completed prior to the transport studies to allow cell phenotype stabilization.^15^ Caco-2 cells between passages 35 and 38 and HT29-MTX-E12 cells between passages 56 and 59 were used in this study.

### 2.4 Characterization of the Caco-2 and HT29-MTX-E12 Cell Monolayers

Caco-2 cell differentiation was evaluated by in situ measurement of alkaline phosphatase (ALP) activity following the procedure of Ferruzza, Rossi, Scarino, and Sambuy (2012).^16^ Mucus production by HT29-MTX-E12 cells was confirmed by Alcian Blue staining following the procedure of Pan, Han, Zhang, Yu, and Liu (2015).^17^ Detailed procedure for these two assays can be found in the Supporting Information.

### 2.5 Evaluation of Cell Viability after Incubation with the 3 kDa Permeates of Faba Bean, Pea, and Soy In Vitro Gastrointestinal Digestates

Cell viability after incubation with the 3 kDa permeate of the legume digestate was verified. Two different concentrations of peptides in the digestate permeate (1120 and 2240 μg of peptide/mL) were investigated. These peptide concentrations were selected to mimic realistic small intestinal exposure. More details on the determination of peptide concentrations can be found in the Supporting Information.

Caco-2 and HT29-MTX-E12 cells (ratio 9:1) were seeded at a final density of 1× 10^5^ cells/cm^2^ in a 96-well black plate with a clear bottom and cultivated for 21 days. Growth media was changed every 2–3 days. After 21 days, growth media was discarded, and cells were washed with 100 μL of D-PBS and then incubated with 100 μL of digestate permeate diluted in HBSS for 2 h at 37 °C to be consistent with the incubation time of the transport experiments. Cells treated with HBSS were used as control of viable cells. After the incubation period, cell viability was measured using the Cell Titer-Glo 2.0 kit (Promega, Fitchburg, Wisconsin), as previously described.^4^ This is a luminescent-based method that quantifies ATP as an indicator of metabolically active cells. The plate was equilibrated to room temperature, and 100 μL of Cell Titer-Glo 2.0 Reagent was added. The plate was shaken for 2 min and incubated 10 min at 22 °C, and luminescence (*L*) was recorded using a Synergy HTX plate reader (Bio-Tek, Winooski, VT, USA). The cell viability was expressed as follows after background subtraction
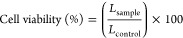
where *L*_sample_ is the luminescence of the sample and *L*_control_ is the luminescence of the cells incubated in HBSS (corresponding to 100% viability).

### 2.6 Transepithelial Transport of Faba Bean, Pea, and Soy Peptides across a Caco-2 and HT29-MTX-E12 Coculture Monolayer

The transport study was conducted following the procedure of Pan, Han, Zhang, Yu, and Liu (2015)^17^ with minor modifications. Caco-2 and HT29-MTX-E12 (ratio 9:1) cells were seeded at a density of 1× 10^5^ cells/cm^2^ in a growth medium on 12 mm polyester transwell inserts with 0.4 μm pore size and cultivated for 21 days. A coculture of Caco-2 and HT29-MTX-E12 cells was selected to mimic more closely the human small intestine by including enterocytes and goblet-like cells. The 9:1 ratio was selected based on enterocyte and goblet cell proportion in the human small intestine.^17−20^ Indeed, in the jejunum, where most peptide absorption takes place,^21^ a ratio of 8–12:1 can be found,^22^ and a 9:1 ratio is generally used in comparable studies.^17,18,23^ The growth medium was changed one last time 24 h before the transport experiment.

On the day of the assay, cells were washed twice with HBSS. Monolayer integrity was evaluated by transepithelial electrical resistance measurement (TEER) with a Millicell ERS-2 Voltohmmeter (EMD Millipore, Billerica, MA) at 37 °C. TEER values were expressed as Ω × cm^2^ and calculated as follows
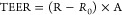
1where *R* is the total measured resistance (Ω), *R*_0_ is the blank resistance (insert without cells), and *A* is the inset effective surface (cm^2^). Only the wells with a TEER > 160 Ω× cm^2^ in HBSS were used for the experiment.^15^ Then, 0.5 mL of faba bean, pea, and soy digestate permeates diluted in HBSS to a final concentration of 2240 μg of peptides/mL was added to the AP side, and 1.5 mL of HBSS was added to the BL side. The cells were incubated for 2 h at 37 °C. At the end of the incubation period, the AP and BL compartment fractions were collected and frozen at −80 °C. The BL fractions were freeze-dried and suspended in 0.5 mL of water to reach the same volume as the AP side. The protein content in the AP and BL fractions after the transport experiment was determined by the pierce BCA protein assay kit using bovine serum albumin as a standard.

Cell monolayer integrity was verified again at the end of the transport experiment by TEER measurement and the Lucifer yellow permeability assay. The latter was measured by adding 0.5 mL of 100 μg/mL Lucifer yellow solution prepared in HBSS to the AP side and 1.5 mL of HBSS to the BL side. The plate was incubated for 1 h at 37 °C, and 150 μL on the BL side was collected and added to a 96-well black plate with clear bottom. Fluorescence was measured (λ_excitation_ = 485 nm, λ_emission_ = 528 nm) using a Synergy HTX microplate reader (Bio-Tek, Winooski, VT) to quantify Lucifer yellow permeability.

### 2.7 Peptide Sequencing by Mass Spectrometry

The peptides present in each fraction (3 kDa permeate of legume digestate, AP and BL compartments) were identified by mass spectrometry as described in Martineau-Côté, Achouri, Wanasundara, Karboune, and L’Hocine (2022).^4^ Briefly, the freeze-dried samples were solubilized in 5% (v/v) acetonitrile and 0.2% (v/v) formic acid and loaded onto a C18 precolumn (0.3 × 5 mm), followed by separation on a reversed-phase column (150 μm × 150 mm) with a linear gradient from 10 to 30% (v/v) acetonitrile and 0.2% (v/v) formic acid at a 600 nL/min flow rate for 56 min using an Ultimate 3000 HPLC system (Eksigent, Dublin, CA, USA) connected to a Q-Exactive Plus mass spectrometer (Thermo Fisher Scientific, San Jose, CA, USA). Each of the full MS spectra that was acquired at a resolution of 70,000 was followed by 12 tandem-MS (MS/MS) spectra on the most abundant multiple-charged precursor ions. The tandem-MS experiments were performed by using collision-induced dissociation at a relative collision energy of 27%. All MS/MS spectra were analyzed using PEAKS Studio (Bioinformatics Solutions, Waterloo, ON Canada; version 10.6) using database searching. PEAKS Studio was set up to search a *V. faba* (faba bean), *Glycine max* (soy), and *Pisum sativum* (pea) database (UniProt/SwissProt). The probability of the identified peptides to be bioactive was predicted in silico using Peptide Ranker.^24^ The physicochemical properties of the identified peptides (isoelectric point, net charge, and hydrophobicity) were calculated using PepDraw (https://www2.tulane.edu/~biochem/WW/PepDraw/).

### 2.8 Antioxidant Activity of 3 kDa Permeates of Legume Digestates and Faba Bean Derived Peptides after Transepithelial Transport

The antioxidant activity of the 3 kDa permeate of the legume digestate before and after transepithelial transport was evaluated by means of the 2.2′-azino-bis (3-ethylbenzothiazoline-6-sulfonic acid) (ABTS) and oxygen radical absorbance capacity (ORAC) assays. The two antioxidant assays were performed as previously described in Martineau-Côté, Achouri, Wanasundara, Karboune, and L’Hocine (2022)^4^ with minor modifications. These assays were also used to measure the antioxidant activity of synthesized faba bean peptides after transepithelial transport.

Briefly, the ABTS assay was performed in preparing a solution containing 7 mM ABTS and 2.45 mM potassium persulfate in water. The solution was incubated overnight to generate free radicals. The solution was diluted in ethanol 100% to reach an absorbance value of 0.70 ± 0.02 at λ = 734 nm. 50 μL of the different peptide fractions (control, AP, and BL) diluted in HBSS (six concentrations ranging from 50 to 1 μg peptides/mL) was mixed with 180 μL of the ABTS solution in a 96-well clear flat-bottom plate and incubated for 6 min at room temperature in the dark. For the synthesized faba bean-derived peptides, 10 peptide concentrations between 10 and 0.05 mM were tested. The absorbance was read at λ = 734 nm with an Epoch microplate spectrophotometer (Bio-Tek, Winooski, VT, USA) microplate reader. The free-radical scavenging capacity was calculated as follows after background subtraction
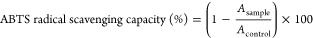
where *A*_sample_ is the absorbance of the sample and *A*_control_ is the absorbance of ABTS in the absence of an antioxidant. The EC_50_ value was reported and was defined as the required peptide concentration to scavenge 50% of the ABTS free radicals. The EC_50_ was calculated using a nonlinear regression with a 4PL curve of the ABTS free-radical scavenging capacity, plotted against its respective peptide concentration.

The ORAC assay was performed following the method of Tomer, McLeman, Ohmine, Scherer, Murray, and O’Neill (2007), as described in Martineau-Côté, Achouri, Wanasundara, Karboune, and L’Hocine (2022).^4^ Twenty-five μL of appropriately diluted peptide fractions (10 μg of peptides/mL for the digestate permeates, AP and BL fractions, 10 μM for synthesized faba bean-derived peptides) and Trolox standard (6.25–50 μM) were loaded in a 96-well black plate with a clear bottom. 150 μL of 96 nM fluorescein solution was added to each well, and the plate was incubated for 30 min at 37 °C. After the incubation period, 25 μL of 79.65 mM 2,2′-azobis(2-amidinopropane) dihydrochloride (AAPH) was added using an automatic injector, and the fluorescence was recorded every minute for 90 min (λ_excitation_ = 485 nm, λ_emission_ = 520 nm) using a Synergy HTX microplate reader (Bio-Tek, Winooski, VT, USA). The area under the curve (AUC) was calculated for the samples, standards, and blanks with the Gen5 Data Analysis Software (BioTek Instruments, Inc., Winooski, VT, USA) using the following regression equation
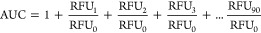
where RFU_0_ is the initial fluorescence and RFU_*x*_ is the relative fluorescence at each time points. Then, the net AUC was calculated as follows
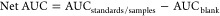


The net AUC value of the Trolox standards was used to build a standard curve. The antioxidant capacity of the samples was expressed as μmol of Trolox equivalents.

### 2.9 Statistical Analysis

Each analysis was performed in triplicate, and results were expressed as mean ± standard deviation (SD). The transepithelial transport experiments were performed on three different days with different passages of cells. Data were analyzed through analysis of variance (ANOVA) (*p* < 0.05), followed by the Tukey’s honest significant difference (HSD) (*p* < 0.05) using the XLSTAT software (Addinsoft, NY) to determine significant differences.

## 3 Results and Discussion

### 3.1 Characterization of the Caco-2 and HT29-MTX-E12 Cell Monolayers

Before conducting the transport experiment, the Caco-2 and HT29-MTX-E12 monolayers were characterized through the measurement of TEER, ALP activity, and Alcian Blue staining to confirm that the Caco-2 and HT29-MTX-E12 cells adopted a differentiated enterocyte and a goblet cell-like phenotype, respectively (Figure S1). As expected, the TEER increased exponentially until the cells reached confluence (7 days). Then, the TEER plateaued for the remaining differentiation period, demonstrating the presence of an intact monolayer. The TEER value after 21 days of culture was in the same range as other studies.^15,17,18,25^ ALP activity increased significantly by 2.7-fold after 14 days (*p* = 0.013) and by 5.2-fold after 21 days (*p* < 0.001), indicating the differentiation of Caco-2 cells. HT29-MTX-E12 effectively produced mucins, as indicated by the blue coloration after Alcian Blue staining. The blue coloration was absent in Caco-2 cells, confirming the absence of mucins. These results demonstrated that the Caco-2 and HT29-MTX-E12 cells adopted a differentiated enterocyte and a goblet-cell-like phenotype, indicating that the formed cell monolayer was representative of the intestinal barrier and was a suitable model for the peptide transport experiments.

### 3.2 Cell Viability and Cell Monolayer Integrity after Incubation with the 3 kDa Permeates of Faba Bean, Pea, and Soy Digestates

Cell viability after a 2 h treatment with the 3 kDa permeates of legume digestate (2240 and 1120 μg peptides/mL) was measured to ensure that the selected peptide doses had no cytotoxic effect. As shown in Figure S2, none of the selected peptide concentration had a significant impact on cell viability (*p* > 0.05). Since both concentrations were representative of a realistic legume serving, as detailed in the Supporting Information, the highest dose was selected to perform the transport experiments. After the transport experiment, the cell monolayer integrity was validated. As shown in Figure S2, the TEER value was unaffected by the 2 h sample exposure (*p* > 0.05) and Lucifer yellow permeability was below 10% and not significantly different from untreated cells (*p* > 0.05), stating that the cell monolayer remained intact after exposure with the legume’s digestate samples.^18^

### 3.3 Transepithelial Transport of Faba Bean, Pea, and Soy Peptides

The peptide recovery (%) in the AP and BL after the transport experiment was evaluated by using the BCA assay to estimate the quantity of transported peptides (Table 1). A peptide recovery ranging from 84 to 90% was obtained for faba bean, pea, and soy, which was in the expected range for this type of experiment.^15^ The unrecovered peptides were possibly hydrolyzed during the experiment. There was no significant difference in peptide transportation for faba bean, pea, and soy (*p* > 0.05), which ranged from 0.6 to 1.2%. This level of transportation was comparable to other studies, although highly variable from one study to another. For instance, in a study by Aiello et al., a recovery of 0.05, 0.02, and 0.009% was obtained for three soy-derived peptides in the BL side,^26^ whereas recoveries of 44.81, 21.68, 9.50, and 5.56% were reported for different casein-derived peptides.^27^ These quantitative discrepancies can be explained by the peptide properties, such as their length, size, amino acid composition, and susceptibility to metabolic degradation and analytical differences.^26^

**Table 1:** Peptides Recovery (%) in the AP and BL Compartments after Cellular Transepithelial Transport Experiment as Assessed with the BCA Assay^a^

	Peptides recovery (%)		
	AP side^b^	BL side^c^	total^d^
Faba Bean			
Fabelle	86.2 ± 7.5^b^	1.2 ± 0.9^b^	87.4 ± 7.3^b^
Malik	86.6 ± 4.7^b^	1.1 ± 0.3^b^	87.7 ± 4.5^b^
Snowbird	86.0 ± 7.4^b^	0.6 ± 0.2^b^	86.6 ± 7.3^b^
Pea			
Amarillo	89.3 ± 7.8^b^	0.8 ± 0.4^b^	90.1 ± 8.1^b^
Soy			
AAC-26-15	82.9 ± 4.9^b^	0.6 ± 0.1^b^	83.5 ± 4.9^b^
*p*-value	0.832	0.346	0.808

a Values are means ± SD of three experiments. Means in a column with a common letter are not significantly different (*p* > 0.05), as analyzed by one-way ANOVA and the Tukey’s test. Peptides recovery (%) was calculated as follows.

b Peptides recovery in the AP compartment = 100(*V*_AP_ × *C*_AP_)/(*V*_Ctrl_ × *C*_Ctrl_).

c Peptides recovery in the BL compartment = 100(*V*_BL_ × *C*_BL_)/(*V*_Ctrl_ × *C*_Ctrl_).

d Total peptide recovery = 100[(*V*_AP_ × *C*_AP_) + (*V*_BL_ × *C*_BL_)]/(*V*_Ctrl_ × *C*_Ctrl_), where *V*_Ctrl_ is the volume added to the AP side at the start of the experiment (*t* = 0 h) and *V*_AP_ and *V*_BL_ are the volumes recovered on each side at the end of the experiment (*t* = 2 h). *C*_ctrl_ is the peptide concentration added to the AP side at the beginning of the experiment (*t* = 0 h) and *C*_AP_ and *C*_BL_ are the peptide concentrations measured in the AP and BL sides at the end of the experiment (*t* = 2 h). All peptide concentrations were measured with the use of the BCA assay, and the peptide content in the AP and BL compartment of cells treated with HBSS was subtracted from the readings

**Figure 2:**
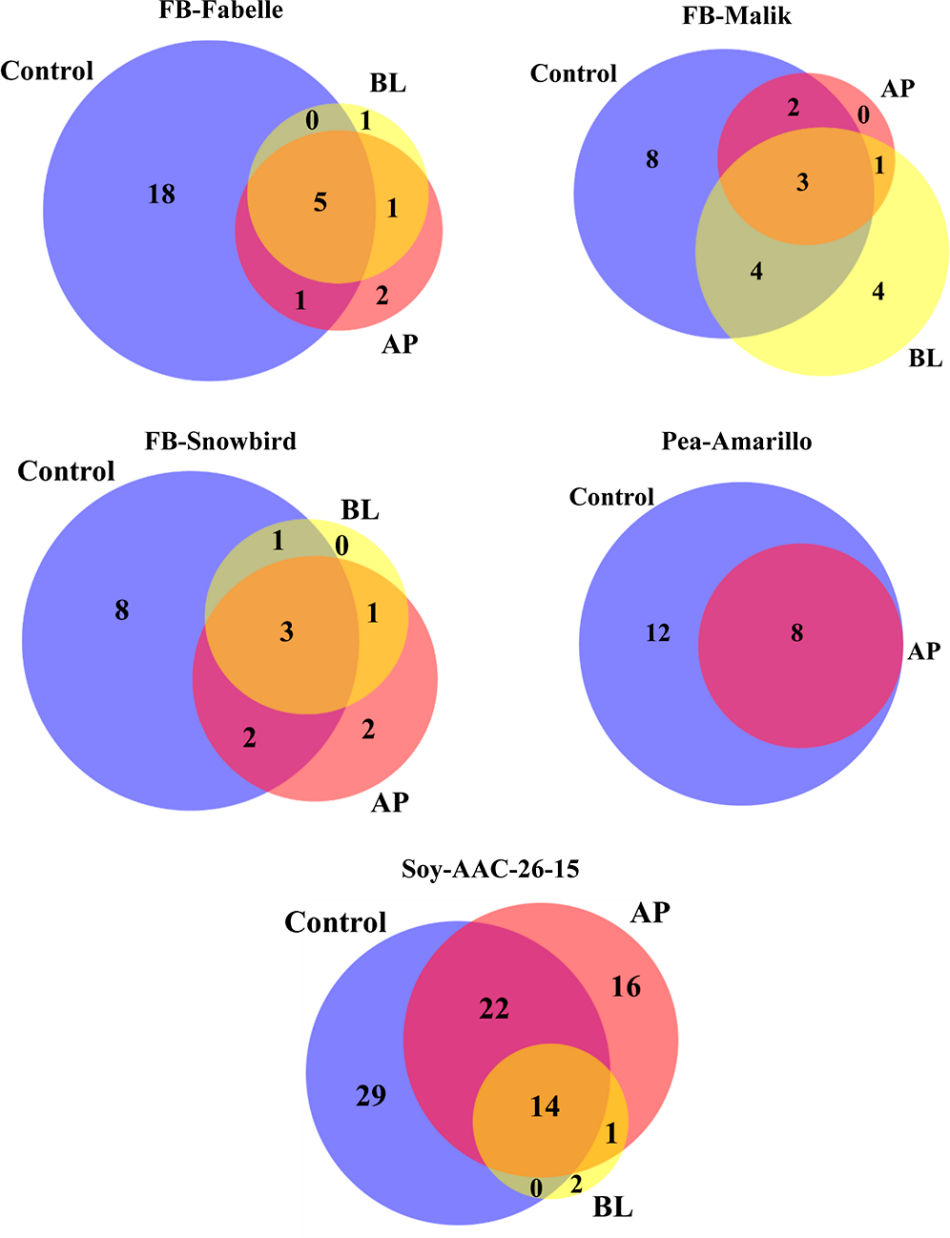
Number of unique peptides identified in the 3 kDa permeates of legume digestates before incubation with the Caco-2 and HT29-MTX-E12 cell monolayers (control) and in the AP and in BL fractions after 2 h of incubation with the intestinal cell monolayer. The blue, pink and yellow circles represent the control and the AP and BL compartments, respectively. Only the peptides found in three independent transport experiments are reported. The proportional Venn diagrams were created using DeepVenn (https://www.deepvenn.com/).

Although peptide transportation was identical on a quantitative level for the three legumes, the profile of the transported peptide might still be different. To evaluate that, the peptides present in the control and the AP and BL fractions were sequenced by mass spectrometry (Table S1). The control was the 3 kDa permeate prior to application to the cell monolayer. As expected, the majority of identified peptides were fragments of globulins, the most abundant storage proteins found in faba bean, pea, and soy,^28−30^ and more specifically of 11S globulins. The data of Table S1 are summarized in Figure 2 to illustrate the number of identified peptides in the control and the AP and BL fractions for the three legumes.

For FB-Fabelle, five peptides (TETWNPNHPEL, TETWNPNHPE, EEEDEDEPR, KEEEDEDEPR, and VVIPTEPPH) were found in common in the control and the AP and BL fractions. For FB-Malik and FB-Snowbird, three peptides were found to be common in each of the three fractions. The presence of these peptides in the three fractions means that they are resistant to brush border peptidases, and a portion of them can be transported intact across the cellular intestinal monolayer. For the three faba bean varieties, one peptide (TETWNPNHP) was found in common in the AP and BL fractions but was absent in the control. This peptide was possibly generated after further hydrolysis by cell membrane brush border peptidases. However, 18, 8, and 8 peptides were only detected in the control for FB-Fabelle, FB-Malik, and FB-Snowbird, respectively, and not in the AP and BL fractions, meaning that they were probably not resistant to the cell membrane peptidases.

The number of transported peptides identified for faba bean (6 for FB-Fabelle, 9 for FB-Malik, and 5 for FB-Snowbird) (Figure 4) was in a similar order of magnitude of other plant-based protein hydrolysates. For instance, 5, 11, and 8 transported peptides were identified in a hempseed peptic hydrolysate,^31^ in a tryptic,^32^ and a pectic lupin hydrolysate.^32^ Still, the number of identified peptides is highly dependent on the digestion method and the analytical workflow used to identify them.

**Figure 3:**
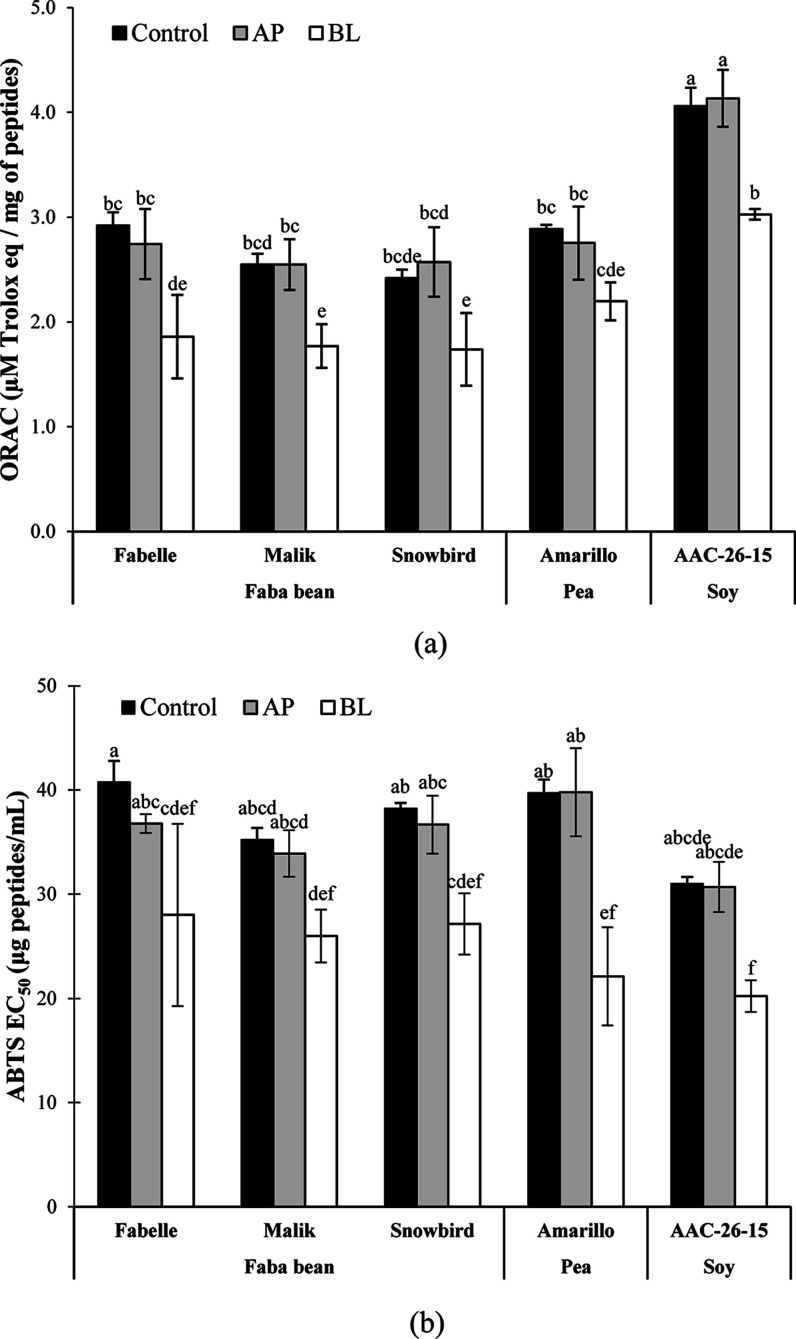
Antioxidant activity of the control and the AP and BL fractions: (a) ORAC assay; (b) ABTS assay. Data are expressed as mean ± SD of three experiments. Means with a common letter are not significantly different (*p* > 0.05), as analyzed by ANOVA and the Tukey’s test.

**Figure 4:**
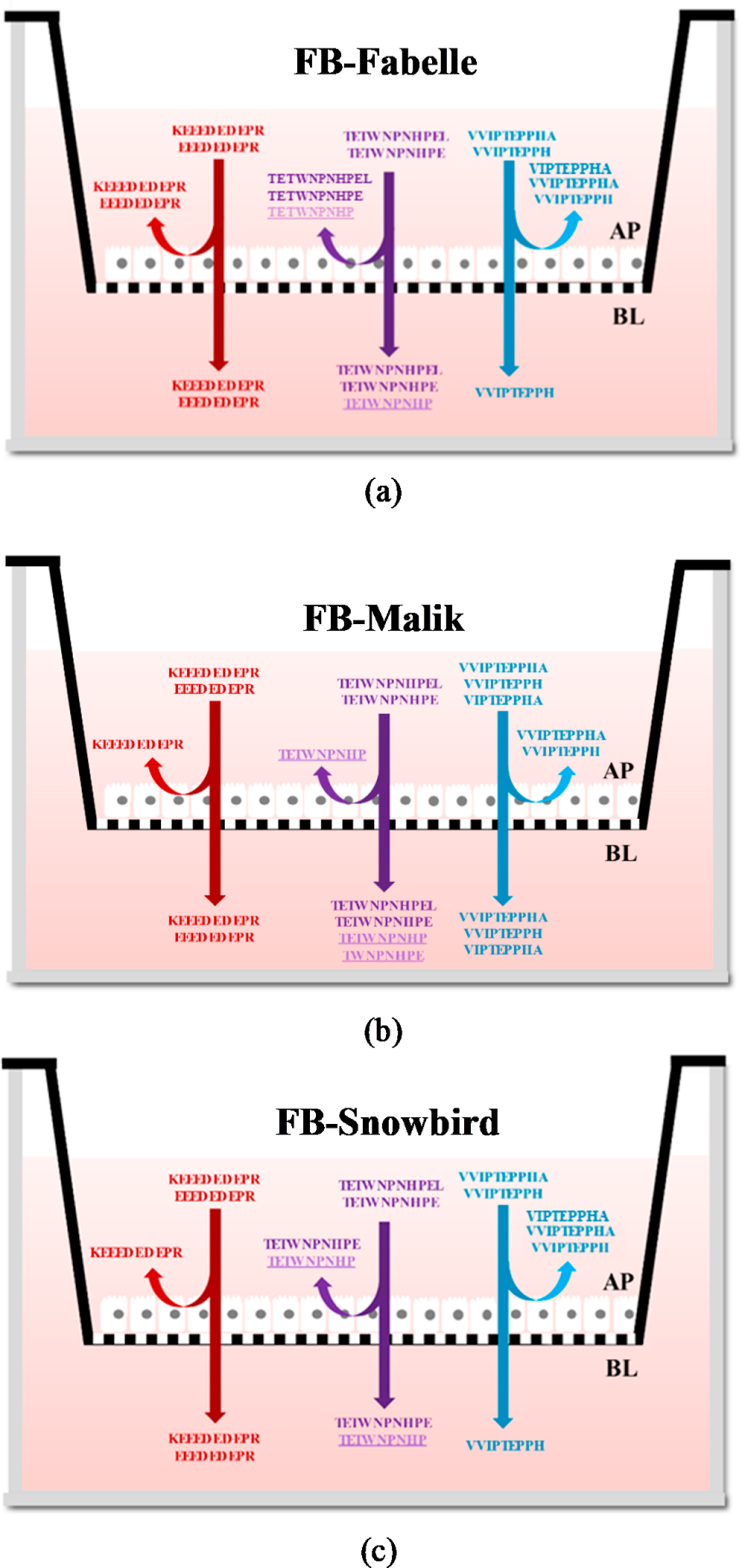
Graphical representation of transported faba bean-derived peptides. Peptide fragments that are coming from the same location on the same parent protein are grouped and in the same color. Peptides generated after exposition to the cell monolayer (metabolites) are underlined and in a lighter font color: (a) FB-Fabelle; (b) FB-Malik; and (c) FB-Snowbird.

In the case of soy, 14 peptides were found in common in the control and the AP and BL fractions. Twenty-nine peptides were present only in the control, and 22 peptides were found in common in the control and in the AP fraction but not in the BL fraction. These peptides are therefore resistant to cell brush border peptidases but were not transported across the cell monolayer. The fact that a higher number of peptides were identified for soy compared to faba bean can be explained by the more extensive soy protein database,^33^ facilitating peptide identification.

In the case of pea, 12 peptides were found only in the control, and 8 peptides were found in common in the control and the AP side. None of them were identified in the BL fraction. This finding was surprising since the recovery data suggested a similar transportation on a quantitative level. This can be explained by the fact that the pea and faba bean proteome is fragmented, which has an impact on peptide identification. Moreover, smaller peptides were possibly not identifiable under the MS experimental conditions used, which represent a limitation of this study. Other authors reported comparable situations. For instance, Corrochano, Ferraretto, Arranz, Stuknytė, Bottani, O’Connor, Kelly, De Noni, Buckin, and Giblin (2019)^34^ did not identify any peptides in the BL fraction when performing transport experiment with an in vitro gastrointestinal digestate of lactoferrin and α-lactalbumin, whereas they identified 14–31 peptides in the BL fractions with those of whey protein isolate, bovine serum albumin, β-lactoglobulin, and a milk protein sport product.

### 3.4 Peptides Structure and Transportability Relationship

The relationship between peptide structure and transportability is still not well understood.^35^ Accordingly, our data demonstrated that faba bean and soy absorbable peptides are highly heterogeneous in terms of length, molecular weight, polarity, and N and C terminal residues (Table S2). For faba bean and soy, the transported peptides had a molecular weight ranging from 960 to 2250 Da and were composed of 8 to 19 residues. These two properties were in the same range as other studies conducted with soy (814–1983 Da),^36^ hempseed (869–1292 Da),^31^ lupin (789–2719 Da),^32^ and casein (<500–1600 Da)^37^ protein hydrolysates.

In terms of hydrophobicity, the values fluctuated between +12.49 and +38.08 kcal/mol for faba bean and between +7.62 and +56.56 kcal/mol for soy peptides, which again compares to other studies. The N- and C-terminal residues are highly heterogeneous for both faba bean and soy, and no patterns can be established, contrary to a defined pattern as reported by Wang, Xie, and Li (2019).^35^ This finding can be explained by the high diversity of digestive proteases and peptidases used to mimic gastrointestinal digestion in this study, leading to a highly diverse peptide profile. Nonetheless, some similarities can still be observed between the transepithelial transported peptides profile for faba bean and soy, where 96% of the absorbable peptides were negatively charged at physiological pH. This is in good agreement with the study of Wang, Xie, and Li (2016),^38^ which demonstrated that negatively charged casein-derived peptides have a higher permeability across Caco-2 cells compared to positively charged ones. Picariello, Ferranti, Fierro, Mamone, Caira, Di Luccia, Monica, and Addeo (2010)^39^ also found that negatively charged peptides had a better resistance to gastrointestinal digestion. Another common trait in faba bean and soy absorbable peptides is the presence of proline residues in their sequence. All of the transported identified peptides possessed at least one proline residue in their sequence, and 64% of them possessed multiple proline residues. This is in good agreement with the fact that proline containing peptides are more resistant to gastrointestinal digestion.^40−42^ However, numerous peptides identified in the control and the AP fractions of faba bean, pea, and soy had a negative net charge and a proline residue but were not transported across the intestinal cell monolayer (Table S1), suggesting that other factors, still to be established, have an impact on peptide transportability.

The physicochemical properties of peptides can also influence the transport routes taken across the intestinal barrier. There are four possible transport routes for peptides, namely, paracellular transport through tight junctions, passive diffusion, transcytosis, and/or intestinal active transporters such as PepT1.^43^ The paracellular route is mostly used by hydrophilic peptides since the tight junctions are a water-filled extracellular route,^35,44^ whereas passive diffusion and transcytosis are preferred by lipophilic peptides. PepT1 is mostly involved in the transport of small di- and tripeptides.^27,45^ Based on this body of knowledge, paracellular transport is probable for the different soy identified peptides, with the exception of QFPFPRPP which is less polar (+7.62 kcal/mol). In the case of faba bean peptides, the paracellular route is more probable for EEEDEDEPR and KEEEDEDEPR, which have multiple negative charges and are highly polar. However, transcytosis and passive diffusion are possible for the other faba bean peptides that are less polar. Further research will be needed to determine the respective mode of peptide transport, through the use of specific inhibitors and/or enhancers of the different uptake pathways.^46^

### 3.5 Antioxidant Activity of Faba Bean, Pea, and Soy In Vitro Gastrointestinal Digestate after Transepithelial Transport

The residual antioxidant activity was measured to evaluate whether the legume digestates were maintaining their activity after transepithelial transport. The antioxidant activity was measured with the 2.2′-azino-bis (3-ethylbenzothiazoline-6-sulfonic acid) (ABTS) and the oxygen radical absorbance capacity (ORAC) assays (Figure 3) since faba bean-derived peptides were previously revealed to have a high activity in these assays.^3,4^ Moreover, these assays are complementary since they measure two different free radical scavenging mechanisms, the former measuring free radical scavenging through single electron transfer and the latter measuring free radical scavenging through hydrogen atom transfer.

For the ORAC assay (Figure 3), there were no significant differences (*p* < 0.05) between the control and the AP fractions for the different legumes, indicating that exposure to intestinal brush border peptidases did not alter the antioxidant activity. However, the activity of the BL side was significantly decreased. This can be explained by several factors, such as the nontransportation or hydrolysis of highly active peptides and/or the transportation of peptides with no activity. Despite having lower ORAC values than the control, the BL fractions still maintained a high residual antioxidant activity of 64% for FB-Fabelle, 69% for FB-Malik, 72% for FB-Snowbird, 76% for pea, and 75% for soy, demonstrating a high potential to maintain an effect in vivo. Besides, it is also important to consider that the 3 kDa permeate of the legume flour digestate contains a mixture of different bioactive molecules such as polyphenols, oligosaccharides, and free amino acids.^4^ In particular, tryptophan, tyrosine, histidine, methionine, and cysteine are important free radical scavengers.^47^ These bioactive molecules may have contributed to some extent to the total antioxidant activity measured. Further research is needed to estimate the exact contribution of each component to the measured antioxidant activity.

For the ABTS assay (Figure 3), a different trend was observed, where the BL fraction had an antioxidant activity higher than that of the control for FB-Fabelle, FB-Snowbird, Pea, and Soy. For FB-Malik, the difference was not statistically significant (*p* = 0.083), although the antioxidant activity also tended to increase. This observed increase could possibly result from an increased transportation of highly active peptides and/or further hydrolysis of peptides to smaller fragments with a higher activity. The different trend observed in the ORAC and ABTS assays can be explained by their respective mechanism of action.^48^

Noteworthily, no significant difference in antioxidant activity was found between soy, pea, and faba beans after transepithelial transport (Figure 3), although a lower EC_50_ was obtained for FB-Malik, FB-Snowbird, and soy compared to FB-Fabelle and pea before transepithelial transport.^4^ This result demonstrates that the transepithelial transport may have an impact on the bioactivity potency. It also reiterates the importance to further confirm peptide bioavailability in in vivo experiments.

### 3.6 Antioxidant Activity of Peptides Derived from Faba Bean In Vitro Gastrointestinal Digestion and Transepithelial Transport

In a previous study,^4^ we have demonstrated that faba bean peptides generated after in vitro gastrointestinal digestion have a high bioactive potential, particularly in terms of antihypertensive and antioxidant activity. These bioactive properties were associated with the release of bioactive peptides after in vitro gastrointestinal digestion.^3^ Seven potent antioxidant peptides were identified, namely, NYDEGSEPR, TETWNPNHPEL, TETWNPNHPE, VIPTEPPH, VIPTEPPHA, VVIPTEPPHA, and VVIPTEPPH (Table S3). These peptides had a strong antioxidant activity, particularly in the ABTS and ORAC assays. Among them, the peptides TETWNPNHPEL and TETWNPNHPE had the highest antioxidant activity, which was 2 to 3 times that of Trolox in the ORAC assay on a molar basis. The peptides VIPTEPPH, VIPTEPPHA, VVIPTEPPHA, and VVIPTEPPH, in addition to being antioxidant, were angiotensin-converting enzyme inhibitors.^3^

Five of these identified faba bean bioactive peptides (TETWNPNHPEL, TETWNPNHPE, VIPTEPPHA, VVIPTEPPHA, and VVIPTEPPH) were transported to the BL compartments, demonstrating their potential to reach the bloodstream and exert their bioactive properties (Figure 4). The peptides EEEDEDEPR and KEEEDEDEPR, which exhibited a low antioxidant activity, were also transported for all faba bean varieties (Table S3). The peptides NYDEGSEPR and VIPTEPPH, which showed high antioxidant activity, were, however, not transported, suggesting a limited bioactive potential in vivo (Table S3). These results could explain the decrease in the measured ORAC activity after transepithelial transport for the three faba bean varieties.

**Figure 5:**
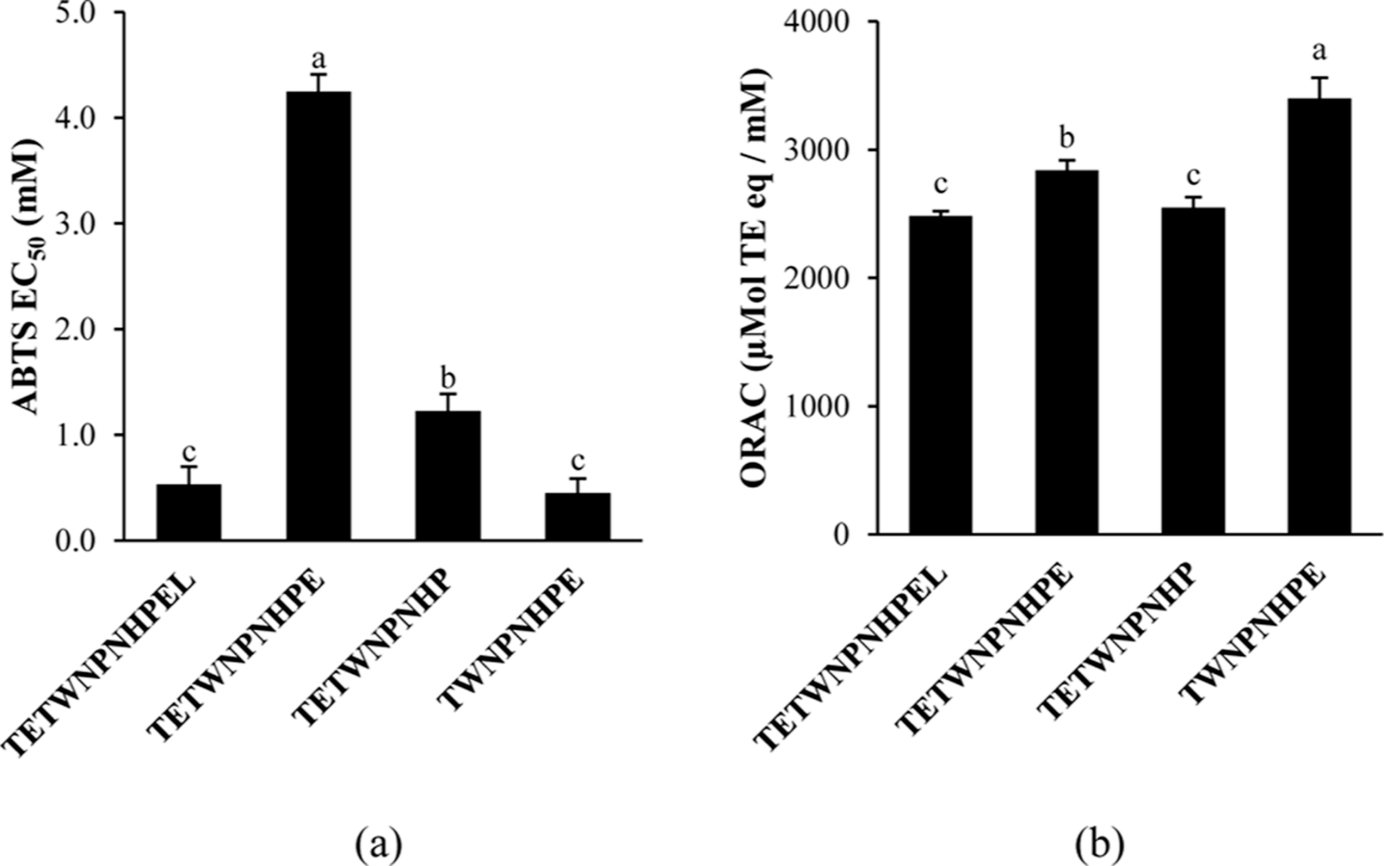
Antioxidant activity of synthesized faba bean peptides derived from in vitro gastrointestinal and transepithelial transport. Data are expressed as mean ± SD of three experiments. Means with a common letter are not significantly different (*p* > 0.05), as analyzed by one-way ANOVA and the Tukey’s test: (a) ABTS assay; (b) ORAC assay.

Interestingly, the peptides TETWNPNHPEL and TETWNPNHPE were partly metabolized after incubation with the intestinal cell monolayer to smaller fragments, TETWNPNHP and TWNPNHPE (Figure 4). The fragment TETWNPNHP was formed for all three faba bean varieties, and the fragment TWNPNHPE was only found in FB-Malik (Figure 4). The formation of these peptide metabolites may have an impact on the antioxidant activity of the BL fractions since TETWNPNHPEL and TETWNPNHPE were previously found to be major contributors of the overall antioxidant activity.^3^ Based on that, the peptide metabolites TETWNPNHP and TWNPNHPE were synthesized to measure their antioxidant activity in comparison to the parent fragments TETWNPNHPEL and TETWNPNHPE (Figure 5). The results showed that the metabolites TETWNPNHP and TWNPNHPE had a significantly higher (*p* < 0.05) activity in the ABTS assay compared to TETWNPNHPE, as demonstrated by their up to 3 times lower EC_50_. The higher activity of TETWNPNHP could have contributed to the increased activity of the 3 kDa permeate of the faba bean digestate after transepithelial transport when assessed with the ABTS assay. With the ORAC assay, TWNPNHPE had a significantly higher activity compared to both TETWNPNHPEL and TETWNPNHPE. However, the metabolite TETWNPNHP had a lower activity compared to TETWNPNHPE (*p* < 0.05) but was not significantly different than TETWNPNHPEL (*p* > 0.05). The formation of TETWNPNHP could therefore explain to a certain extent the observed decrease in the total activity of the 3 kDa permeate of the faba bean digestate after transepithelial transport in the ORAC assay.

This study has demonstrated for the first time that faba bean peptides generated after in vitro simulated human gastrointestinal digestion are bioaccessible in a cellular model. These peptides can be transported intact across the intestinal barrier while maintaining their antioxidant activity. There were no differences in peptide transportability on a quantitative level between faba bean, pea, and soy, but there were differences in terms of peptide profile and activity potency. Nine faba bean peptides crossed the intestinal cells to the BL compartment, and five of them, namely, TETWNPNHPEL, TETWNPNHPE, VIPTEPPHA, VVIPTEPPH, and VVIPTEPPHA, were antioxidants. Interestingly, the most potent antioxidant peptides, TETWNPNHPEL and TETWNPNHPE, were metabolized to smaller fragments (TETWNPNHP and TWNPNHPE). These two metabolites revealed to be potent antioxidants, contributing to the preservation of the antioxidant activity after transepithelial transport.

Based on the results of this study, it can be inferred that the benefits of faba bean protein consumption are likely to go beyond fulfilling nutritional needs. The presence of bioaccessible bioactive peptides after gastrointestinal digestion may contribute to its health benefits, in complementarity to dietary fibers, resistant starch, polyphenols, and other bioactives. Although being very promising for their bioactive potential, the transportation of large faba bean-derived oligopeptides across the intestinal epithelium may raise issue on their allergenicity potential.^49^ Data on this aspect are lacking and will require further assessment.

Currently, the health benefits related to bioactive peptides remain controversial since peptides produced through nonphysiological enzymatic hydrolysis often failed to maintain their alleged bioactive activities in vivo due to gastrointestinal digestion and/or poor permeability across the intestinal barrier. The present study has, however, for the first time investigated the bioactivity of peptides from faba bean proteins in the context of physiologically relevant in vitro human gastrointestinal digestion and absorption models. Moreover, this study has considered the release of bioactive peptides from the digestion of heat-treated flours of three different faba bean varieties, soy and pea, therefore taking into account the impact of the food matrix and processing, and the genotype. A limitation of this study is, however, the absence of a digestion blank to compensate for digestive enzyme contributions to the measured bioactive properties of the legume digestates. The assessment of the digestive enzyme contribution remains a complex task because of the increased enzymatic autolysis occurring in the absence of a substrate.^14^ Research is ongoing to find the most appropriate way to compensate for the digestive enzyme contribution without overestimating it.^14,50−52^

In future investigations, the use of cellular antioxidant assays and/or the AOP1 assay in HepG2 cells^53^ could also be considered to confirm the antioxidant activity of the BL fractions in a more physiologically relevant manner. Moreover, the present human intestinal membrane model could be further improved by including other cell-types to mimic M-cells and immune cells, for instance, but would require a thorough validation. Lastly, in vivo and clinical human studies are still needed to confirm the present results and get a better insight on the health benefits of faba bean protein and its potential use as a biofunctional food ingredient.

## Supporting Information Available

Additional experimental details and results; characterization of the Caco-2 and HT29-MTX co-culture monolayer; cell viability and monolayer integrity after incubation with the 3 kDa permeate of legume digestates; faba bean, pea, and soy peptides identified in the control (3 kDa permeate of legume digestate before the transport experiment) and in the AP and the BL fractions at the end of the transepithelial transport experiment; physicochemical properties and in silico predicted bioactive fragments of transported faba bean and soy peptides; and antioxidant activity of peptides derived from faba bean flour in vitro gastrointestinal digestion and transepithelial transport (PDF)
