# Supplementary material for: Bioaccessibility and Antioxidant Activity of Faba Bean Peptides in Comparison to those of Pea and Soy after In Vitro Gastrointestinal Digestion and Transepithelial Transport across Caco-2 and HT29-MTX-E12 Cells
Source: J Agric Food Chem. 2024 Aug 1;72(32):17953–63. doi: 10.1021/acs.jafc.4c02948 (PMC11328180; doi:10.1021/acs.jafc.4c02948)
Supplement: Supplementary file 1 — jf4c02948_si_001.pdf [file jf4c02948_si_001.pdf]

Supporting Information For:

Bioaccessibility and Antioxidant Activity of Faba Bean Peptides in Comparison to those of Pea and Soy after In Vitro Gastrointestinal Digestion and Transepithelial Transport across Caco-2 and HT29-4 MTX-E12 Cells

Delphine Martineau-Côté[1,2], Allaoua Achouri[1], Melanie Pitre[1], Salwa Karboune[2] and Lamia L'Hocine[1,*]

[1]Agriculture and Agri-Food Canada, Saint-Hyacinthe Research and Development Centre, Saint-Hyacinthe, Quebec J2S 8E3, Canada

[2]Department of Food Science and Agricultural Chemistry, Macdonald Campus, McGill University, Sainte-Anne-de-Bellevue, Quebec H9X 3V9, Canada

*Email: lamia.lhocine@agr.gc.ca. Phone: 1-514-726-0718.

TABLE OF CONTENTS

1. ADDITIONAL METHODOLOGY DETAILS 3

1.1 ALKALINE PHOSPHATASE (ALP) ACTIVITY 3

1.2 ALCIAN BLUE STAINING 4

1.3 SELECTION OF THE PEPTIDE CONCENTRATION FROM LEGUMES *IN VITRO* GASTROINTESTINAL DIGESTATE FOR TRANSEPITHELIAL TRANSPORT STUDIES 4

2. SUPPLEMENTARY FIGURES 5

Figure S1. Characterization of the Caco-2 and HT29-MTX co-culture monolayer 5

Figure S2. Cell viability and monolayer integrity after incubation with the 3 kDa permeate of legume digestates. 6

3. SUPPLEMENTARY TABLES 7

Table S1. Faba bean, pea and soy peptides identified in the control (3 kDa permeate of legume digestate before the transport experiment) and in the apical (AP) and the basolateral (BL) fractions at the end of the transepithelial transport experiment 7

Table S2. Physicochemical properties and in silico predicted bioactive fragments of transported legume peptides. 14

Table S3. Antioxidant Activity of Peptides Derived from Faba Bean Flour *in vitro* Gastrointestinal Digestion and Transepithelial Transport 15

4. REFERENCES 16

1. Additional Methodology Details

1.1 Alkaline Phosphatase (ALP) Activity

Caco-2 and HT29-MTX-E12 (ratio 9:1) cells were seeded at a density of 1×10^5 cells/cm^2 in growth medium on transwell inserts. On the day of the assay, growth medium was discarded, and cells were washed twice with PBS containing 1 mM $CaCl_2$ and 1 mM $MgCl_2$ (PBS+) on the apical and basolateral sides. All solutions were pre-warmed to 37 °C. Reaction buffer was prepared in mixing a 2.5 mg/mL p-nitrophenyl phosphate (p-NPP) solution containing 100 mM diethanolamine, 150 mM NaCl and 2 mM $MgCl_2$ with a 10 mM Tris-HCl buffer at pH 8.0 containing 150 mM NaCl (ratio 3:1). 1.0 mL of the reaction buffer was added to the apical side and 1.0 mL of PBS+ to the basolateral side and the plate was incubated at 37 °C. 100 µL of the reaction buffer in the apical side was collected after 0, 2, 4, 6 and 8 minutes of reaction and added to a 96 well microplate plate kept on ice. The wells of the microplate contained 50 µL of 0.5 N NaOH to stop the reaction. Absorbance was recorded at λ=405 nm with an Epoch microplate spectrophotometer (Bio-Tek, Winooski, VT, USA) and a standard curve was built with 2-nitrophenol (p-NP). Absorbance was converted to concentration and p-NP concentration in the test sample was plotted as a function of time. The slope of the linear curve was used to calculate ALP activity. ALP activity was expressed as mU, where 1 mU represents the release of 1 nmol of p-NP per minute at 37 °C. Alkaline phosphatase activity was normalized to cellular protein content. To do so, cells were scrapped on ice in RIPA buffer to provoke cell lysis and transferred to a micro-centrifuge tube. The tube was incubated at 4 °C with shaking for 30 minutes and then centrifuged at 16,000 x g for 20 minutes at 4 °C. Protein content in the supernatant was determined with the Pierce BCA protein assay kit using bovine serum albumin as standard.

1.2 Alcian Blue Staining

Briefly, Caco-2 (negative control), Caco-2 and HT29-MTX-E12 co-culture (ratio 9:1) and HT29-MTX-E12 cells were seeded at a final density of 1× 10^5 cells/cm^2 in growth medium on transwell inserts and cultivated for 21 days. After 21 days, growth medium was discarded, and cells were washed twice with PBS. Cells were fixed with a 4% formaldehyde solution prepared in PBS for 30 minutes at room temperature. Cells were washed again twice with PBS and once with 1% acetic acid. Cells were stained for 30 minutes at room temperature with a 1% alcian blue solution prepared in 3% acetic acid. Cells were washed twice with PBS and visualized with a ZEISS Primovert inverted microscope (Carl Zeiss Microscopy, Oberkochen, Germany).

1.3 Selection of Peptide Concentration from the Legumes *in vitro* Gastrointestinal Digestate for Transepithelial Transport Studies

Peptide concentration used for the transepithelial transport experiment was selected to mimic potential intestinal exposure in a realistic manner as described by Mahler, Esch, Tako, Southard, Archer, Glahn and Shuler (2012) [1] and Zhang, Zhang, Ma and Cui (2020) [2]. The protein nutritional need for an adult is 0.8 g per kg of body weight per day [3]. The consumption of 50 up to 100 g of dried pulse is accepted as a realistic serving in a healthy diet [4]. This serving size represents on average 15–30 g of proteins for faba bean, assuming a 30% protein content. The small intestine has a surface area of ~200 m^2 [5]. Most of dietary peptide absorption takes place in the jejunum [6], which as a surface of ~60 m^2 [7]. Therefore, the consumption of 50–100 g of dried faba bean will lead to a potential exposure of ~25–50 µg protein/cm^2 in the jejunum. The transwell insert used in the experiment (12 wells) has a growth surface of 1.12 cm^2. However, the microvilli structure increases the surface area by a factor 20 [8]. The real absorption surface was therefore 22.4 cm^2 per insert. Thus, the addition of 0.5 mL of a solution of 1120–2240 µg of peptides/mL per wells led to the targeted concentration.

2. Supplementary Figures

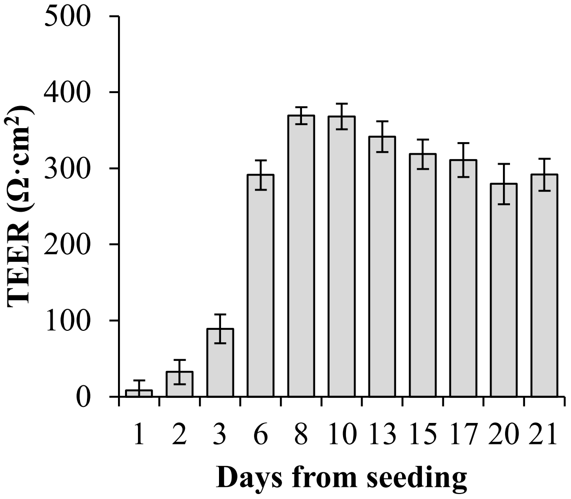

(a)

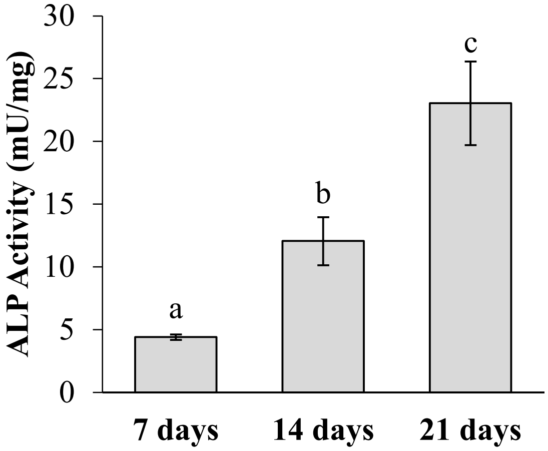

(b)

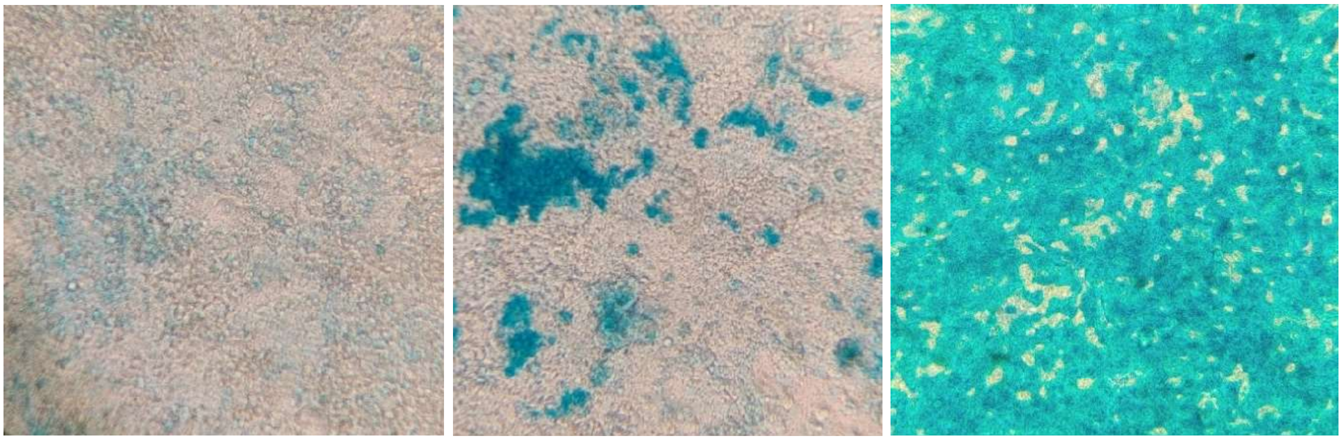

Caco-2 **Caco2 and HT29-MTX (ratio 9:1)** **HT29-MTX**

(c)

Figure S1. Characterization of the Caco-2 and HT29-MTX co-culture monolayer; (a) Transepithelial electrical resistance (TEER) was measured over time to evaluate cell monolayers integrity. Data are means ± standard deviation of 12 monolayers of one representative experiment; (b) Caco-2 cell differentiation was evaluated by *in situ* measurement of alkaline phosphatase activity (ALP). Data are expressed as Mean ± standard deviation of three experiments and means without a common letter differ ($p < 0.05$) as analyzed by one-way ANOVA and the Tukey's test; (c) mucus production by HT29-MTX cells was confirmed by means of alcian blue staining and visualization was performed with an inverted phase microscope (20x magnification). The presence of a blue coloration indicates the presence of intestinal mucins.

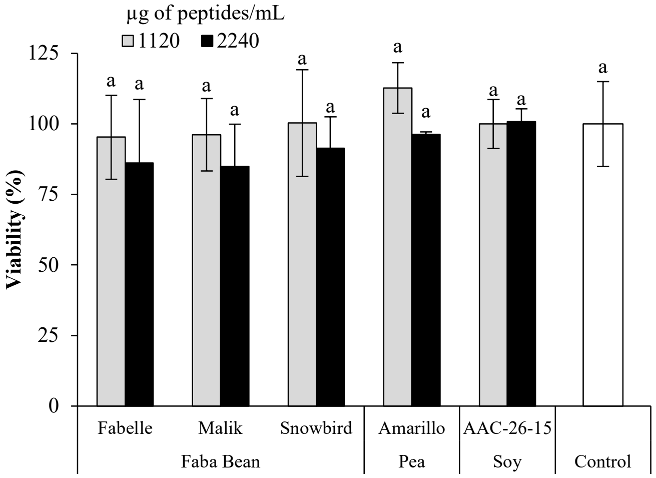

(a)

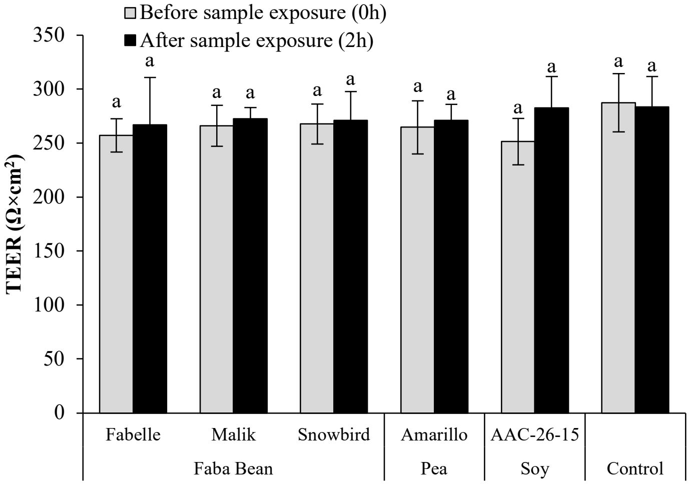

(b)

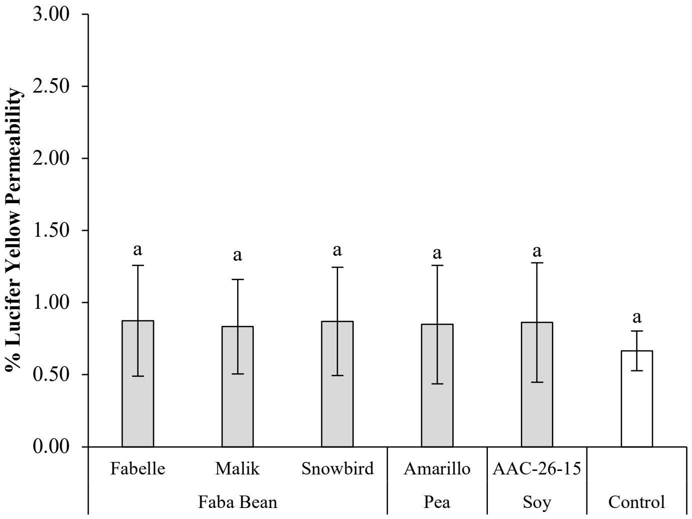

(c)

Figure S2. Cell viability and monolayer integrity after incubation with the 3 kDa permeate of legume digestates. Data are means ± standard deviation of three experiments performed on three different days. Means with a common letter are not significantly different ($p > 0.05$) as analyzed by ANOVA and the Tukey's test; (a) Cell viability was assessed with the Cell Titer-Glo 2.0 kit, which quantifies ATP as an indicator of metabolically active cells. The results are expressed as a percentage of untreated cells; (b) TEER was measured in HBSS before the addition of the 3 kDa permeates of legume digestate (T=0) and after the 2-hours incubation period with the 3 kDa permeates of legume digestate (2240 µg of peptides/mL); (c) Lucifer yellow permeability was measured after the 2-hours incubation with the 3 kDa permeates of legume digestate (2240 µg of peptides /mL).

3. Supplementary Tables

Table S1. Faba bean, pea and soy peptides identified in the control (3 kDa permeate of legume digestate before the transport experiment) and in the apical (AP) and the basolateral (BL) fractions at the end of the transepithelial transport experiment

Fabelle (Faba bean)

Peptides[1]	Control	AP	BL	MH+ [Da]	Accession Number	Fragment Location	Precursor Protein	Organism	Peptide Ranker Score[2]
EDEDEDEKEEQ	+	-	-	1394.5080	CAA81262.1	*306–316*	Legumin	*Vicia faba*	0.04
EDEDEDEKEEQEQ	+	-	-	1651.6092	CAA81262.1	*306-318*	Legumin	*Vicia faba*	0.03
**EEEDEDEPR**	**+**	**+**	**+**	**1147.4387**	**CAA81262.1**	***327-335***	**Legumin**	***Vicia faba***	**0.08**
ETWNPNHP	-	+	-	994.4377	CAA81262.1	*53-60*	Legumin	*Vicia faba*	0.37
**KEEEDEDEPR**	**+**	**+**	**+**	**1275.5337**	**CAA81262.1**	***326-335***	**Legumin**	***Vicia faba***	**0.07**
SEKEDEDEDEKEEQ	+	-	-	1738.6776	CAA81262.1	*303-316*	Legumin	*Vicia faba*	0.04
SQGEEEEEEER	+	-	-	1350.5292	CAA81262.1	*283-293*	Legumin	*Vicia faba*	0.05
**TETWNPNHP**	**-**	**+**	**+**	**1095.4854**	**CAA81262.1**	***52-60***	**Legumin**	***Vicia faba***	**0.22**
**TETWNPNHPE**	**+**	**+**	**+**	**1224.5280**	**CAA81262.1**	***52-61***	**Legumin**	***Vicia faba***	**0.15**
**TETWNPNHPEL**	**+**	**+**	**+**	**1337.6121**	**CAA81262.1**	***52-62***	**Legumin**	***Vicia faba***	**0.27**
QEEDEDEDEDEKEE	+	-	-	1767.6202	CAA38757.1	*262-275*	Legumin A1 pre-pro-polypeptide	*Vicia faba*	0.04
QEEDEDEDEDEKEER	+	-	-	1923.7213	CAA38757.1	*262-276*	Legumin A1 pre-pro-polypeptide	*Vicia faba*	0.05
QEEDEDEDEKE	+	-	-	1394.5080	CAA38758.1	*271-281*	Legumin A2 primary translation product	*Vicia faba*	0.05
QEEDEDEDEKEE	+	-	-	1523.5506	CAA38758.1	*271-282*	Legumin A2 primary translation product	*Vicia faba*	0.05
QEEDEDEDEKEER	+	-	-	1679.6517	CAA38758.1	*271-283*	Legumin A2 primary translation product	*Vicia faba*	0.07
QEEEEEEEEEK	+	-	-	1436.5548	CAA27313.1	*285-295*	Legumin B	*Vicia faba*	0.04
QQQPDSHQ	+	-	-	967.4229	CAA27313.1	*127-134*	Legumin B	*Vicia faba*	0.13
VIPTEPPHA	-	+	-	960.5150	CDQ12453.1	*155-163*	Tonoplast intrinsic protein 32	*Vicia faba*	0.25
**VVIPTEPPH**	**+**	**+**	**+**	**988.5463**	**CDQ12453.1**	***154-162***	**Tonoplast intrinsic protein 32**	***Vicia faba***	**0.19**
VVIPTEPPHA	+	+		1059.5834	CDQ12453.1	*154-163*	Tonoplast intrinsic protein 32	*Vicia faba*	0.19
NYDEGSEPR	+	-	-	1066.4436	AAA33660.1	*30-38*	Convicilin	*Pisum sativum*	0.14
NQLDSTPR	+	-	-	930.4640	CAA47809.1	*173-180*	Legumin	*Pisum sativum*	0.24
EDVPNHGT	+	-	-	868.3796	CAA34906.1	*141-148*	Lipoxygenase-2	*Pisum sativum*	0.20
GGSSTHPYP	+	-	-	902.4003	CAA34906.1	*233-241*	Lipoxygenase-2	*Pisum sativum*	0.52
NDLGNPDHGEH	+	-	-	1204.4979	CAA34906.1	*216-226*	Lipoxygenase-2	*Pisum sativum*	0.22
LGNPDSGENH	+	-	-	1039.4440	CAA30666.1	*213-222*	Lipoxygenase-3	*Pisum sativum*	0.19
NDLGNPDSGENH	-	-	+	1268.5139	CAA30666.1	*211–222*	Lipoxygenase-3	*Pisum sativum*	0.16
KEEDEEDEPR	+	-	-	1275.5337	CAW45393.1	*13–22*	Unnamed protein product	*Glycine max*	0.07

Table S1. (continued).

Peptides[1]	Control	AP	BL	MH+ [Da]	Accession Number	Fragment Location	Precursor Protein	Organism	Peptide Ranker Score[2]
**Malik (Faba bean)**									
EDEDEDEKEEQ	+	+	-	1394.5080	CAA81262.1	*306–316*	Legumin	*Vicia faba*	0.04
**KEEEDEDEPR**	**+**	**+**	**+**	**1275.5337**	**CAA81262.1**	***326-335***	**Legumin**	***Vicia faba***	**0.07**
**EEEDEDEPR**	**+**	**-**	**+**	**1147.4387**	**CAA81262.1**	***327-335***	**Legumin**	***Vicia faba***	**0.08**
QEEDEDEDEKEE	-	-	+	1523.5506	CAA38758.1	*271-282*	Legumin A2 primary translation product	*Vicia faba*	0.05
QEEDEDEDEKEER	+	-	-	1679.6517	CAA38758.1	*271-283*	Legumin A2 primary translation product	*Vicia faba*	0.07
QEEEEEEEEEK	+	+	-	1436.5548	CAA27313.1	*285-295*	Legumin B	*Vicia faba*	0.04
SEKEDEDEDEKEEQ	+	-	-	1738.6776	CAA81262.1	*303-316*	Legumin	*Vicia faba*	0.04
SQGEEEEEEER	+	-	-	1350.5292	CAA81262.1	*283-293*	Legumin	*Vicia faba*	0.05
**TETWNPNHP**	**-**	**+**	**+**	**1095.4854**	**CAA81262.1**	***52-60***	**Legumin**	***Vicia faba***	**0.22**
**TETWNPNHPE**	**+**	**-**	**+**	**1225.5121**	**CAA81262.1**	***52-61***	**Legumin**	***Vicia faba***	**0.15**
**TETWNPNHPEL**	**+**	**-**	**+**	**1337.6121**	**CAA81262.1**	***52-62***	**Legumin**	***Vicia faba***	**0.27**
**TWNPNHPE**	**-**	**-**	**+**	**994.4377**	**CAA81262.1**	***54-61***	**Legumin**	***Vicia faba***	**0.31**
**VIPTEPPHA**	**+**	**-**	**+**	**960.5150**	**CDQ12453.1**	***155-163***	**Tonoplast intrinsic protein 32**	***Vicia faba***	**0.25**
**VVIPTEPPH**	**+**	**+**	**+**	**988.5463**	**CDQ12453.1**	***154-162***	**Tonoplast intrinsic protein 32**	***Vicia faba***	**0.19**
**VVIPTEPPHA**	**+**	**+**	**+**	**1059.5834**	**CDQ12453.1**	***154-163***	**Tonoplast intrinsic protein 32**	***Vicia faba***	**0.19**
IGANEPSEH	+	-	-	953.4323	CAA61947.1	*150-158*	Fructose-1,6-bisphosphate aldolase	*Pisum sativum*	0.11
LGNPDSGENH	+	-	-	1039.4440	CAA30666.1	*213-222*	Lipoxygenase-3	*Pisum sativum*	0.19
NDLGNPDHGEH	+	-	-	1204.4979	CAA34906.1	*216-226*	Lipoxygenase-2	*Pisum sativum*	0.22
NYDEGSEPR	-	-	+	1067.4277	AAA33660.1	*30-38*	Convicilin	*Pisum sativum*	0.14
YDEGSEPR	-	-	+	952.4007	AAA33660.1	*31–38*	Convicilin	*Pisum sativum*	0.16
KEEDEEDEPR	+	-	-	1275.5337	CAW45393.1	*13–22*	Unnamed protein product	*Glycine max*	0.07
QFPFPRPP	+	-	-	985.5255	BAA23360.2	*152–159*	Alpha subunit of beta conglycinin	*Glycine max*	0.97

Table S1. (continued)

**Snowbird (Faba bean)**									
**Peptides[1]**	**Control**	**AP**	**BL**	**MH+ [Da]**	**Accession Number**	**Fragment Location**	**Precursor Protein**	**Organism**	**Peptide Ranker Score[2]**
EDEDEDEKEEQ	-	+	-	1,394,508	CAA81262.1	306–316	Legumin	*Vicia faba*	0.04
**EEEDEDEPR**	**+**	**-**	**+**	**1147.4387**	**CAA81262.1**	**327-335**	**Legumin**	***Vicia faba***	**0.08**
**KEEEDEDEPR**	**+**	**+**	**+**	**1275.5337**	**CAA81262.1**	**326-335**	**Legumin**	***Vicia faba***	**0.07**
QEEDEDEDEDEKEE	+	-	-	1767.6202	CAA38757.1	262-275	Legumin A1 pre-pro-polypeptide	*Vicia faba*	0.04
QEEDEDEDEKEE	+	-	-	1523.5506	CAA38758.1	271-282	Legumin A2 primary translation product	*Vicia faba*	0.05
QEEDEDEDEKEER	+	-	-	1679.6517	CAA38758.1	271-283	Legumin A2 primary translation product	*Vicia faba*	0.07
QEEEEEEEEEK	+	+	-	1436.5548	CAA27313.1	285-295	Legumin B	*Vicia faba*	0.04
QQQPDSHQ	+	-	-	967.4229	CAA27313.1	127-134	Legumin B	*Vicia faba*	0.13
SEKEDEDEDEKEEQ	+	-	-	1738.6776	CAA81262.1	303-316	Legumin	*Vicia faba*	0.04
**TETWNPNHP**	**-**	**+**	**+**	**1095.4854**	**CAA81262.1**	**52-60**	**Legumin**	***Vicia faba***	**0.22**
**TETWNPNHPE**	**+**	**+**	**+**	**1224.528**	**CAA81262.1**	**52-61**	**Legumin**	***Vicia faba***	**0.15**
TETWNPNHPEL	+	-	-	1337.6121	CAA81262.1	52-62	Legumin	*Vicia faba*	0.27
VIPTEPPHA	-	+	-	960.515	CDQ12453.1	155-163	Tonoplast intrinsic protein 32	*Vicia faba*	0.25
**VVIPTEPPH**	**+**	**+**	**+**	**988.5463**	**CDQ12453.1**	**154-162**	**Tonoplast intrinsic protein 32**	***Vicia faba***	**0.19**
VVIPTEPPHA	+	+	-	1059.5834	CDQ12453.1	154-163	Tonoplast intrinsic protein 32	*Vicia faba*	0.19
NDLGNPDHGEH	+	-	-	1204.4979	CAA34906.1	216–226	Lipoxygenase-2	*Pisum sativum*	0.22
KEEDEEDEPR	+	-	-	1275.5337	CAW45393.1	13–22	Unnamed protein product	*Glycine max*	0.07

Table S1. (continued)

**Amarillo (Pea)**									
**Peptides[1]**	**Control**	**AP**	**BL**	**MH+ [Da]**	**Accession Number**	**Fragment Location**	**Precursor Protein**	**Organism**	**Peptide Ranker Score[2]**
SDDEDTAPPR	+	-	-	1102.4649	BAB32793.1	973–982	110 kDa 4SNc-Tudor domain	*Pisum sativum*	0.35
NYDEGSEPR	+	-	-	1066.4436	AAA33660.1	30–38	Convicilin	*Pisum sativum*	0.14
GDGMPGGGSNGSGPGPK	+	+	-	1444.6126	AAA82975.1	627–643	Heat shock protein hsp70	*Pisum sativum*	0.73
QEEDEDEEK	+	-	-	1150.4384	CAA10722.1	250-258	LegA class precursor	*Pisum sativum*	0.04
QEEDEDEEKQPR	+	-	-	1531.6509	CAA10722.1	250-261	LegA class precursor	*Pisum sativum*	0.09
QEEEEDEDEER	+	+	-	1436.5297	CAA10722.1	268-278	LegA class precursor	*Pisum sativum*	0.05
RGEEEEEDKKE	+	-	-	1377.613	CAA10722.1	286-296	LegA class precursor	*Pisum sativum*	0.04
KEDEDEDEEEE	+	-	-	1395.492	CAA47809.1	308-318	Legumin	*Pisum sativum*	0.04
KEDEDEDEEEEE	+	-	-	1524.5346	CAA47809.1	308-319	Legumin	*Pisum sativum*	0.04
KEDEDEDEEEEEE	+	+	-	1653.5772	CAA47809.1	308-320	Legumin	*Pisum sativum*	0.04
KEDEDEDEEEEEER	+	-	-	1809.6783	CAA47809.1	308-321	Legumin	*Pisum sativum*	0.05
HSEKEEEDEDEPR	+	-	-	1628.6672	CAA47809.1	328-340	Legumin	*Pisum sativum*	0.05
SEKEEEDEDEPR	+	-	-	1491.6083	CAA47809.1	329–340	Legumin	*Pisum sativum*	0.06
EEEDEDEPR	+	+	-	1147.4387	CAA47809.1	332–340	Legumin	*Pisum sativum*	0.08
REEEEEEEEDEEK	+	+	-	1708.6669	S26688	299–311	Legumin K	*Pisum sativum*	0.03
EEEEEEEEDEEK	+	-	-	1552.5658	S26688	300-311	Legumin K	*Pisum sativum*	0.03
NDLGNPDHGEH	+	+	-	1204.4979	CAA34906.1	216-226	Lipoxygenase-2	*Pisum sativum*	0.22
NDLGNPDSGENH	+	+	-	1268.5139	CAA30666.1	211-222	Lipoxygenase-3	*Pisum sativum*	0.16
VVIPTEPPHA	+	-	-	1059.5834	CDQ12453.1	154-163	Tonoplast intrinsic protein 32	*Vicia faba*	0.19
VVIPTEPPH	+	+	-	988.5463	ACU23484.1	237–245	Unknown	*Glycine max*	0.19

Table S1. (continued)

**AAC-26-15 (Soy)**									
**Peptides[1]**	**Control**	**AP**	**BL**	**MH+ [Da]**	**Accession Number**	**Fragment Location**	**Precursor Protein**	**Organism**	**Peptide Ranker Score[2]**
EGKDEDEEEEGH	+	+	-	1402.5243	AAB71140.1	*82-93*	2S albumin pre-pro-peptide	*Glycine max*	0.08
DEDEDEEQDER	+	-	-	1408.4985	BAA23360.2	*141-151*	Alpha subunit of β-conglycinin	*Glycine max*	0.06
EEDEDEQPRP	+	+	-	1243.5075	BAA23360.2	*94-103*	Alpha subunit of β-conglycinin	*Glycine max*	0.15
**EEDEDEQPRPI**	**+**	**+**	**+**	**1356.5916**	**BAA23360.2**	***94-104***	**Alpha subunit of β-conglycinin**	***Glycine max***	**0.14**
EEDEDEQPRPIP	+	+	-	1453.6444	BAA23360.2	*94-105*	Alpha subunit of β-conglycinin	*Glycine max*	0.15
EPQQPGEKEEDEDEQPR	+	+	-	2039.8792	BAA23360.2	*86-102*	Alpha subunit of β-conglycinin	*Glycine max*	0.11
**EPQQPGEKEEDEDEQPRPI**	**+**	**+**	**+**	**2250.0161**	**BAA23360.2**	***86-104***	**Alpha subunit of β-conglycinin**	***Glycine max***	**0.12**
GSEEEDEDEDEEQDER	+	+	-	1939.6798	BAA23360.2	*136-151*	Alpha subunit of β-conglycinin	*Glycine max*	0.05
**KEEDEDEQPRPI**	**-**	**+**	**+**	**1484.6866**	**BAA23360.2**	***93-104***	**Alpha subunit of β-conglycinin**	***Glycine max***	**0.11**
KQEEDEDEEQQ	+	+	-	1406.5556	BAA23360.2	*166-176*	Alpha subunit of β-conglycinin	*Glycine max*	0.03
**QFPFPRPP**	**+**	**+**	**+**	**985.5255**	**BAA23360.2**	***152-159***	**Alpha subunit of β-conglycinin**	***Glycine max***	**0.97**
QFPFPRPPH	+	-	-	1122.5844	BAA23360.2	*152-160*	Alpha subunit of β-conglycinin	*Glycine max*	0.92
GVMNGGMQPR	+	-	-	1047.4712	BAA03681.1	*371-380*	Basic 7S globulin	*Glycine max*	0.32
MNGGMQPR	+	-	-	891.3813	BAA03681.1	*373-380*	Basic 7S globulin	*Glycine max*	0.48
VMDKPNGPV	+	+	-	956.4871	BAA03681.1	*343-351*	Basic 7S globulin	*Glycine max*	0.28
EDEGEQPRP	+	-	-	1056.4594	ADD38965.1	*94-102*	β-conglycinin alpha’ subunit	*Glycine max*	0.21
EDQDEDEEQDKE	+	-	-	1508.551	ADD38965.1	*179-190*	β-conglycinin alpha’ subunit	*Glycine max*	0.05
EDQDEDEEQDKESQ	+	-	-	1723.6416	ADD38965.1	*179-192*	β-conglycinin alpha’ subunit	*Glycine max*	0.04
**EEDEGEQPRP**	**+**	**+**	**+**	**1185.502**	**ADD38965.1**	***93-102***	**β-conglycinin alpha’ subunit**	***Glycine max***	**0.15**
**EEDEGEQPRPFP**	**-**	**-**	**+**	**1429.6232**	**ADD38965.1**	***93-104***	**β-conglycinin alpha’ subunit**	***Glycine max***	**0.29**
EEEDQDEDEEQDKE	-	+	-	1766.6362	ADD38965.1	*177-190*	β-conglycinin alpha’ subunit	*Glycine max*	0.05
ESEEEEEDQDEDEEQDKE	+	-	-	2240.796	ADD38965.1	*173-190*	β-conglycinin alpha’ subunit	*Glycine max*	0.03
**GEKEEDEGEQPRP**	**+**	**+**	**+**	**1499.6611**	**ADD38965.1**	***90-102***	**β-conglycinin alpha’ subunit**	***Glycine max***	**0.13**
**KEEDEGEQPRP**	**-**	**-**	**+**	**1313.597**	**ADD38965.1**	***92-102***	**β-conglycinin alpha’ subunit**	***Glycine max***	**0.13**
TEVGPDDDEK	+	-	-	1104.4694	AAB03894.1	*329-338*	Glucose binding protein	*Glycine max*	0.07
AGNPDIEHPET	+	+	-	1179.5278	BAA19058.1	*166-176*	Glycinin	*Glycine max*	0.28
**DEDDEDEQIPSHPP**	**+**	**+**	**+**	**1622.6456**	**BAA74953.1**	***287-300***	**Glycinin**	***Glycine max***	**0.16**
DEDDEDEQIPSHPPR	-	+	-	1778.7467	BAA74953.1	*287-301*	Glycinin	*Glycine max*	0.23
DEDEDDEDEQIPSHPP	-	+	-	1866.7152	BAA74953.1	*285-300*	Glycinin	*Glycine max*	0.12
DEDEDEDEDDEDEQIPSHPP	+	+	-	2354.8544	BAA74953.1	*281-300*	Glycinin	*Glycine max*	0.07
**DEDEDEDEDKPRPS**	**+**	**+**	**+**	**1675.6569**	**BAA74953.1**	***311-324***	**Glycinin**	***Glycine max***	**0.11**

Table S1. (continued)

AAC-26-15 (Soy)									
**Peptides[1]**	**Control**	**AP**	**BL**	**MH+ [Da]**	**Accession Number**	**Fragment Location**	**Precursor Protein**	**Organism**	**Peptide Ranker Score[2]**
**DEDEDEDEDQPR**	+	+	+	**1491.5357**	**BAA74953.1**	***338-349***	**Glycinin**	***Glycine max***	**0.08**
DEDEDEDKPRPS	+	-	-	1431.5873	BAA74953.1	*313-324*	Glycinin	*Glycine max*	0.16
DEDEDEDQPR	+	-	-	1247.4661	BAA74953.1	*340-349*	Glycinin	*Glycine max*	0.13
DEDEQIPSHPP	+	+	-	1263.549	BAA74953.1	*290-300*	Glycinin	*Glycine max*	0.27
DEDEQIPSHPPR	+	+	-	1419.6501	BAA74953.1	*290-301*	Glycinin	*Glycine max*	0.39
**DQDEDEDEDEDQPR**	+	+	+	**1734.6213**	**BAA74953.1**	***336-349***	**Glycinin**	***Glycine max***	**0.08**
**DQDQDEDEDEDEDQPR**	+	+	+	**1977.7069**	**BAA74953.1**	***334-349***	**Glycinin**	***Glycine max***	**0.07**
EDDEDEDEEEDQPRPD	-	+	-	1961.7007	BAA19058.1	*282-297*	Glycinin	*Glycine max*	0.06
EDDEDEDEEEDQPRPDHPPQRPS	+	+	-	2761.1097	BAA19058.1,	*282-304*	Glycinin	*Glycine max*	0.90
EDDEDEQIPSHPP	-	+	-	1507.6186	BAA74953.1	*288-300*	Glycinin	*Glycine max*	0.18
EDEDEDEDKPRPS	+	-	-	1560.6299	BAA74953.1	*312-324*	Glycinin	*Glycine max*	0.13
EDEDEDEDQPR	+	-	-	1376.5087	BAA74953.1	*339-349*	Glycinin	*Glycine max*	0.10
EDQPRPDHPPQRPSRPEQ	-	+	-	2166.0438	BAA19058.1	*291-308*	Glycinin	*Glycine max*	0.24
EEDQPRPDHPPQ	+	-	-	1444.6454	BAA19058.1	*290-301*	Glycinin	*Glycine max*	0.26
EEDQPRPDHPPQRPSRPEQ	+	-	-	2295.0864	BAA19058.1	*290-308*	Glycinin	*Glycine max*	0.20
EEEDQPRPDHPPQRPSRPEQ	+	-	-	2424.129	BAA19058.1	*289-308*	Glycinin	*Glycine max*	0.17
**EQDEDEDEDEDKPR**	+	+	+	**1748.6733**	**BAA74953.1**	***309-322***	**Glycinin**	***Glycine max***	**0.07**
**EQDEDEDEDEDKPRPS**	+	+	+	**1932.7581**	**BAA74953.1**	***309-324***	**Glycinin**	***Glycine max***	**0.09**
**EQDQDQDEDEDEDEDQPR**	+	+	+	**2234.8081**	**BAA74953.1**	***332-349***	**Glycinin**	***Glycine max***	**0.05**
EQTPSYPPR	+	+	-	1074.5215	BAA19058.1	*266-274*	Glycinin	*Glycine max*	0.49
GNPDIEHPET	-	+	-	1108.4907	BAA19058.1	*167-176*	Glycinin	*Glycine max*	0.31
HEDDEDEDEEEDQPR	+	+	-	1886.6798	BAA19058.1	*281-295*	Glycinin	*Glycine max*	0.05
**HEDDEDEDEEEDQPRPD**	+	+	+	**2098.7596**	**BAA19058.1**	***281-297***	**Glycinin**	***Glycine max***	**0.05**
HEDDEDEDEEEDQPRPDHPPQRPS	-	+	-	2898.1686	BAA19058.1	*281-304*	Glycinin	*Glycine max*	0.90
HEDDEDEDEEEDQPRPDHPPQRPSRPE	+	-	-	3280.3651	BAA19058.1	*281-307*	Glycinin	*Glycine max*	0.92
HEDDEDEDEEEDQPRPDHPPQRPSRPEQ	-	+	-	3408.4237	BAA19058.1	*281-308*	Glycinin	*Glycine max*	0.92
LAGNPDIEHPET	+	+	-	1292.6119	BAA19058.1	*165-176*	Glycinin	*Glycine max*	0.26
NNQLDQNPR	+	-	-	1099.5128	BAA19058.1	*153-161*	Glycinin	*Glycine max*	0.25
QDEDEDEDEDQPR	+	-	-	1619.5943	BAA74953.1	*337-349*	Glycinin	*Glycine max*	0.08
REQDEDEDEDEDKPR	+	-	-	1904.7744	BAA74953.1	*308-322*	Glycinin	*Glycine max*	0.06
REQDEDEDEDEDKPRPS	+	-	-	2088.8592	BAA74953.1	*308-324*	Glycinin	*Glycine max*	0.07
EEEEEDEKPQ	+	+	-	1261.5068	AAA33966.1	*280-289*	Glycinin G1	*Glycine max*	0.04
EEEEEEDEKPQ	+	-	-	1390.5494	AAA33966.1	*279-289*	Glycinin G1	*Glycine max*	0.04

Table S1. (continued)

Peptides[1]	Control	AP	BL	MH+ [Da]	Accession Number	Fragment Location	Precursor Protein	Organism	Peptide Ranker Score[2]
**AAC-26-15 (Soy)**									
EEEEEEEDEKPQ	+	+	-	1519.592	AAA33966.1	*278-289*	Glycinin G1	*Glycine max*	0.04
VIKPPTDEQQQRPQ	-	+	-	1663.8765	AAA33966.1	*264-277*	Glycinin G1	*Glycine max*	0.10
KEEEEEEESKKEEEE	+	-	-	1909.8034	KAG4386526.1	*421-435*	Hypothetical protein	*Glycine max*	0.03
GDDDMPGAGGAGSGAGPK	-	+	-	1516.6337	KAG5063899.1	*630-647*	Hypothetical protein	*Glycine max*	0.76
GVANPEETHPK	+	-	-	1178.5801	KAG4944389.1	*91-101*	Hypothetical protein	*Glycine max*	0.17
NSWDPPNPH	-	+	-	1063.4592	AAA33983.1	*130–138*	Lectin pre-peptide	*Glycine max*	0.77
SWDPPNPH	-	+	-	949	AAA33983.1	*131-138*	Lectin pre-peptide	*Glycine max*	0.86
NDLGDPDKGENH	+	-	-	1310.561	AAB41272.1	*208-219*	Lipoxygenase-3	*Glycine max*	0.19
ADTGGGDAVRPV	+	+	-	1156.5595	AAB71226.1	*2-13*	Metallothionein-II protein	*Glycine max*	0.17
ADTSGGDAVRPV	-	+	-	1186.57	AAB65792.1	*2-13*	Metallothionein-II protein	*Glycine max*	0.16
SGGDAVRPV	+	-	-	857.4476	AAB65792.1	*5-13*	Metallothionein-II protein	*Glycine max*	0.37
EHAMNPVPQ	+	-	-	1038.4674	ACU18271.1	*20–28*	Unknown	*Glycine max*	0.24
FVDLEPTVIDEVRTGT	-	+	-	1790.9173	ACU19580.1	*67–82*	Unknown	*Glycine max*	0.12
KGPDPTPGKPM	+	+	-	1124.5772	ACU13726.1	*14–24*	Unknown	*Glycine max*	0.85
SGDVWFPQPAPK	+	+	-	1328.6635	ACU18647.1	*309–320*	Unknown	*Glycine max*	0.83
VIPTEPPHQ	+	+	-	1017.5365	ACU23484.1	*238–246*	Unknown	*Glycine max*	0.18
VVIPTEPPH	+	+	-	988.5463	ACU23484.1	*237–245*	Unknown	*Glycine max*	0.19
VVIPTEPPHQ	+	-	-	1116.6049	ACU23484.1	*237–246*	Unknown	*Glycine max*	0.13
SADDFEPPLIPPK	+	-	-	1425.7263	CAW94712.1	*216–228*	Unnamed protein product	*Glycine max*	0.72
NALEPDHRVE	-	+	-	1179.5753	CAA47809.1	*39–48*	Legumin	*Pisum sativum*	0.15

Peptides identified by Q-Exactive MS/MS in the 3 kDa permeate of legumes digestate before the exposition to the cell monolayer (control) and in the apical (AP) and basolateral (BL) fractions at the end of the transport experiment. Only the peptides found in the apical and basolateral fractions of three independent transport experiments are reported. (+) indicates that the peptide was detected and (-) indicates that the peptide was not detected. [1] Amino acid sequence is abbreviated with one letter code. [2]The probability scores of peptides to be bioactive was computed using peptide ranker (Mooney et al., 2012). A score close to 0 indicate that the peptide has a low probability to be bioactive whereas a score close to 1 indicate a high probability to be bioactive.

Table S2. Physicochemical properties and *in silico* predicted bioactive fragments of transported faba bean and soy peptides.

**Transported Peptides**	**Physicochemical Properties**[a]					**Occurrence Frequency of Bioactive Fragments (*in silico* Predicted)**[b]									
**Sequence**[c]	**Length**	**MW (Da)**	**PI**	**Net charge**	**Hydrophobicity (Kcal× mol^{-1})**	**1**	**2**	**3**	**4**	**5**	**6**	**7**	**8**	**9**	**10**
Faba Bean															
EEEDEDEPR	9	1147	3.27	-6	+35.28	0.11	0.11	0.11	-	-	-	0.33	-	-	-
KEEEDEDEPR	10	1275	3.64	-5	+38.08	0.20	0.20	0.10	-	-	-	0.30	-	-	-
TETWNPNHP	9	1095	5.06	-1	+14.25	0.89	0.22	0.11	0.11	-	-	-	-	-	-
TETWNPNHPE	10	1224	4.07	-2	+17.88	0.80	0.20	0.20	0.10	-	-	-	-	0.10	-
TETWNPNHPEL	11	1337	4.07	-2	+16.63	0.73	0.18	0.18	0.36	-	-	-	-	0.09	-
TWNPNHPE	8	994	5.06	-1	+14.00	0.75	0.13	0.25	0.13	-	-	-	-	0.13	-
VIPTEPPHA	9	960	5.06	-1	+13.45	0.89	0.56	-	0.11	-	-	-	-	0.11	-
VVIPTEPPH	9	988	5.06	-1	+12.49	0.89	0.56	-	-	-	-	-	-	0.11	-
VVIPTEPPHA	10	1059	5.06	-1	+12.99	0.90	0.50	-	0.10	-	-	-	-	0.10	-
Soy															
DEDDDEDEQIPSHPP	14	1623	3.22	-7	+36.21	0.43	0.29	0.07	-	-	-	-	-	0.07	-
DEDEDEDEDKPRPS	14	1676	3.44	-7	+45.97	0.21	0.21	0.07	0.07	-	-	-	-	-	-
DEDEDEDEDQPR	12	1491	3.11	-8	+43.34	0.17	0.17	0.08	-	-	-	-	-	-	-
DQDEDEDEDEDQPR	14	1735	3.05	-9	+47.75	0.29	0.14	0.07	-	-	-	-	-	-	-
DQDQDEDEDEDEDQPR	16	1978	3.00	-10	+52.16	0.29	0.14	0.07	-	-	-	-	-	-	-
EEDEDEQPRPI	11	1356	3.34	-5	+31.44	0.27	0.27	0.09	-	-	-	0.09	-	-	-
EEDEGEQPRP	10	1185	3.46	-4	+30.07	0.40	0.50	0.20	-	-	-	0.10	-	-	-
EPQQPGEKEEDEDEQPRPI	19	2250	3.56	-6	+44.47	0.58	0.47	0.11	-	0.05	0.05	0.05	0.05	-	-
EQDEDEDEDEDKPR	14	1749	3.40	-8	+49.77	0.25	0.14	0.06	0.06	-	-	-	-	-	-
EQDEDEDEDEDKPRPS	16	1933	3.40	-8	+50.37	0.25	0.19	0.06	0.06	-	-	-	-	-	-
EQDQDQDEDEDEDEDQPR	18	2235	2.98	-11	+56.56	0.39	0.11	0.06	-	-	-	-	-	-	-
GEKEEDEGEQPRP	13	1500	3.76	-4	+37.65	0.54	0.62	0.23	-	-	-	0.08	-	-	-
HEDDEDEDEEEDQPRPD	17	2099	3.29	-11	+56.71	0.24	0.18	0.06	-	-	0.06	0.18	-	-	-
KEEDEDEQPRPI	12	1485	3.72	-4	+34.24	0.33	0.33	0.08	-	-	-	0.08	-	-	-
KEEDEGEQPRP	11	1313	3.87	-3	+32.87	0.45	0.55	0.18	-	-	-	0.09	-	-	-
QFPFPRPP	8	985	11.6	1	+7.62	0.75	0.88	0.25	-	-	-	-	-	0.13	0.13

Transported peptides found in common in the three faba bean varieties (FB-Fabelle, FB-Malik and FB-Snowbird) are underlined. [a]Physicochemical properties of the transported peptides were predicted using the PepDraw software (http://www.tulane.edu/~biochem/WW/PepDraw/) (PI : Isoelectric point) [b]The occurrence frequency of bioactive fragments in the bioavailable faba bean and soy peptide sequences were predicted *in silico* with the BIOPEP-UWM database (Minkiewicz et al., 2019) (1: DPP-IV inhibitor, 2: ACE inhibitor, 3: DPP-III inhibitor, 4: Antioxidant, 5: Antiamnestic, 6: Antithrombotic, 7: Stimulating vasoactive substance release, 8: Regulating the stomach mucosal membrane activity, 9: α-glucosidase inhibitor, 10: Renin inhibitor). The frequency of occurrence data are shaded proportionally (a darker color means a higher frequency or occurrence). [c]Amino acid sequences are abbreviated with one letter code.

Table S3. Antioxidant Activity of Peptides Derived from Faba Bean Flour *in vitro* Gastrointestinal Digestion and Transepithelial Transport

	Antioxidant Activity[4]		Transported Peptides		
	ORAC (µM Trolox eq / mM)	ABTS EC_{50} (mM)	FB-Fabelle	FB-Malik	FB-Snowbird
NYDEGSEPR	557 ± 5	2.8 ± 0.5	-	-	-
PVNRPGEPQ	6.0 ± 0.1	-	-	-	-
LDNINALEPDH	57 ± 1	-	-	-	-
TETWNPNHPEL	2482 ± 39	0.5 ± 0.2	+	+	-
TETWNPNHPE	2838 ± 78	4.2 ± 0.2	+	+	+
EEEDEDEPR	2.0 ± 0.1	-	+	+	+
KEEEDEDEPR	3.0 ± 0.3	-	+	+	+
VIPTEPPH	56 ± 2	5.7 ± 0.3	-	-	-
VIPTEPPHA	47 ± 3	3.7 ± 0.1	-	+	-
VVIPTEPPHA	49 ± 2	2.9 ± 0.1	-	+	-
VVIPTEPPH	59 ± 1	2.8 ± 0.2	+	+	+
Trolox	.	*0.064 ± 0.005*			

4. References

(1) Mahler, G. J.; Esch, M. B.; Tako, E.; Southard, T. L.; Archer, S. D.; Glahn, R. P.; Shuler, M. L. Oral exposure to polystyrene nanoparticles affects iron absorption. *Nature Nanotechnology* **2012**, *7* (4), 264-271. DOI: 10.1038/nnano.2012.3.
(2) Zhang, R.; Zhang, Q.; Ma, L. Q.; Cui, X. Effects of Food Constituents on Absorption and Bioaccessibility of Dietary Synthetic Phenolic Antioxidant by Caco-2 Cells. *Journal of Agricultural and Food Chemistry* **2020**, *68* (16), 4670-4677. DOI: 10.1021/acs.jafc.9b07315.
(3) Richter, M.; Baerlocher, K.; Bauer, J. M.; Elmadfa, I.; Heseker, H.; Leschik-Bonnet, E.; Stangl, G.; Volkert, D.; Stehle, P.; on behalf of the German Nutrition Society Revised Reference Values for the Intake of Protein. *Ann Nutr Metab* **2019**, *74* (3), 242-250. DOI: 10.1159/000499374 PubMed.
(4) Willett, W.; Rockström, J.; Loken, B.; Springmann, M.; Lang, T.; Vermeulen, S.; Garnett, T.; Tilman, D.; DeClerck, F.; Wood, A.; et al. Food in the Anthropocene: the EAT-Lancet Commission on healthy diets from sustainable food systems. *Lancet (London, England)* **2019**, *393* (10170), 447-492. DOI: 10.1016/s0140-6736(18)31788-4 From NLM.
(5) DeSesso, J. M.; Jacobson, C. F. Anatomical and physiological parameters affecting gastrointestinal absorption in humans and rats. *Food and Chemical Toxicology* **2001**, *39* (3), 209-228. DOI: 10.1016/S0278-6915(00)00136-8 WorldCat.org.
(6) Picariello, G.; Ferranti, P.; Addeo, F. Use of brush border membrane vesicles to simulate the human intestinal digestion. *Food Research International* **2016**, *88*, 327-335. DOI: https://doi.org/10.1016/j.foodres.2015.11.002.
(7) Kararli, T. T. Comparison of the gastrointestinal anatomy, physiology, and biochemistry of humans and commonly used laboratory animals. *Biopharmaceutics & Drug Disposition* **1995**, *16* (5), 351-380. DOI: 10.1002/bdd.2510160502 WorldCat.org.
(8) Campbell, J.; Berry, J.; Liang, Y. Chapter 71 - Anatomy and Physiology of the Small Intestine. In *Shackelford's Surgery of the Alimentary Tract, 2 Volume Set (Eighth Edition)*, Yeo, C. J. Ed.; Content Repository Only!, 2019; pp 817-841.
